# Mechanistic Perspectives From Genomics and Pangenomics of Medicinal and Aromatic Plants: Linking Genome Architecture to Phytochemical Diversity

**DOI:** 10.1155/ijog/2966459

**Published:** 2026-09-15

**Authors:** Himanshu Saini, Jyoti Yadav, Vishal Johar, Shalu Vyas, Vikrant Jaryan, Atin Kumar, Deepak Nanda, Anand Kumar, Jeevan Jyoti Kaushik, Sahil Shamkuwar, Shubham Singh

**Affiliations:** ^1^ School of Applied Natural Science, Adama Science and Technology University, Adama, Ethiopia, astu.edu.et; ^2^ School of Agricultural Sciences, Shri Guru Ram Rai University, Dehradun, Uttarakhand, India; ^3^ School of Agriculture, Galgotias University, Greater Noida, India, galgotiasuniversity.edu.in; ^4^ School of Agriculture, Lovely Professional University, Phagwara, Punjab, India, lpu.in; ^5^ University Institute of Agriculture, Sant Baba Bhag Singh University, Jalandhar, Punjab, India, sbbsuniversity.in; ^6^ School of Agriculture, Uttaranchal University, Dehradun, Uttarakhand, India, uttaranchaluniversity.ac.in; ^7^ Tula′s Institute of Pharmacy, Dehradun, Uttarakhand, India; ^8^ Faculty of Agricultural Sciences, GLA University, Mathura, Uttar Pradesh, India, gla.ac.in; ^9^ School of Basic & Applied Sciences, Shri Guru Ram Rai University, Dehradun, Uttarakhand, India; ^10^ Department of Genetics and Plant Breeding, Institute of Agricultural Sciences, Banaras Hindu University, Varanasi, Uttar Pradesh, India, bhu.ac.in; ^11^ Plant Breeding Graduate Program, University of Florida, Gainesville, Florida, USA, ufl.edu

**Keywords:** artificial intelligence, biosynthetic gene clusters, mechanistic genomics, metabolic engineering, multiomics integration, pangenomics

## Abstract

Medicinal and aromatic plants (MAPs) produce a remarkable diversity of specialized metabolites with significant pharmaceutical, nutraceutical, and industrial value. Although advances in long‐read sequencing, chromosome‐scale genome assembly, and pangenomics have greatly expanded genomic resources, the mechanistic links between genome architecture and phytochemical diversity remain incompletely understood. The present review synthesizes current evidence describing how structural genomic variation may contribute to phytochemical diversity, while acknowledging that many proposed genome‐to‐metabolite relationships require further experimental validation. Examples illustrate how genome architecture is associated with specialized‐metabolite biosynthesis through multiple regulatory processes. However, the strength of supporting evidence varies considerably among MAP species. Moreover, relatively few genome‐to‐metabolite relationships have been confirmed through direct functional validation. We further discuss how pangenomics, multiomics integration, genome editing, synthetic biology, and artificial intelligence support the discovery, validation, and engineering of specialized metabolic pathways. Casual conclusions are evaluated according to the strength of available evidence, highlighting where causal relationships have been experimentally established and where conclusions remain primarily association‐based. Overall, this review provides an integrated conceptual and evidence‐based perspective summarizing proposed relationships between genome architecture and phytochemical diversity and outlines future priorities for functional genomics, precision breeding, metabolic engineering, and sustainable utilization of MAPs.

## 1. Introduction

Medicinal and aromatic plants (MAPs) produce a remarkable diversity of specialized metabolites that have shaped traditional medicine and modern pharmacology for centuries. Beyond their pharmaceutical importance, many MAPs are increasingly recognized within the “food‐medicine homology” framework, where nutritional and therapeutic functions converge. Recent studies on *Artemisia* species demonstrated substantial variation in flavonoids, antioxidant capacity, and medicinal metabolites across developmental stages, emphasizing how growth dynamics influence both nutritional quality and pharmacological potential [1]. Similarly, comprehensive analyses of food‐medicine homologous resources identified diverse phytochemicals, including flavonoids, triterpenes, alkaloids, and polysaccharides. These compounds regulate lipid metabolism through AMPK‐ and PPAR*γ*‐associated pathways, highlighting the translational significance of MAP‐derived metabolites for metabolic disorders such as hyperlipidemia [2].

This diversity spans multiple taxonomic lineages and metabolite classes, including benzylisoquinoline alkaloids in *Papaver* and *Nelumbo*, monoterpenes and phenylpropanoids in *Mentha* and *Ocimum*, diterpenoids in *Salvia* and *Taxus*, triterpenoid saponins in *Panax* and *Glycyrrhiza*, and iridoids/withanolides in *Withania* and *Rauvolfia*. MAP genomes now span numerous medicinal plant families, including Lamiaceae (*Salvia, Ocimum, Mentha*), Rutaceae (*Citrus*), Orchidaceae (*Dendrobium*), Apocynaceae (*Rauvolfia*), Zingiberaceae (*Curcuma*), Papaveraceae (*Papaver*), Cannabaceae (*Cannabis*), and Araliaceae (*Panax*). Together, these genomic resources demonstrate that specialized metabolite evolution has occurred independently across multiple phylogenetic lineages rather than being restricted to only a few model species.

Despite their economic and therapeutic significance, MAPs have historically received limited genomic investigation compared with model plants and major crops. This disparity has begun to narrow with the rapid expansion of long‐read sequencing and chromosome‐scale assembly technologies, which have generated over 400 genome assemblies representing more than 200 MAP species [3]. Genome architecture is increasingly recognized as a major contributor to specialized‐metabolite diversity, although the underlying causal mechanisms have been experimentally validated in only a limited number of MAP species.

Unlike most food crops, MAPs frequently exhibit extensive diversification of lineage‐specific specialized metabolic pathways. Many of these pathways are associated with structurally dynamic genomic regions containing tandem duplications, transposable elements (TEs), and biosynthetic gene clusters (BGCs). Therefore, understanding MAP genomes requires more than assembling chromosomes; it requires explaining how genome architecture has evolved to coordinate the synthesis of pharmacologically active compounds that frequently display strong tissue specificity, developmental regulation, and environmental responsiveness. These biological characteristics distinguish MAP genomics from conventional crop genomics and justify the need for mechanistic genomic approaches.

MAP genomes pose distinct challenges that limit functional interpretation. Many species possess extremely large and repetitive genomes enriched in TEs, which disrupt assembly contiguity and obscure the evolutionary origins of biosynthetic gene families [4]. High heterozygosity, segmental duplications, and recurrent whole‐genome duplication (WGD) events further complicate haplotype resolution and accurate gene annotation [5, 6]. These structural features intersect with the biology of specialized metabolism, which is often governed by multigene BGCs that exhibit strong tissue‐specific, developmental, and environmental regulation [7]. As a result, linking gene content to metabolic function remains challenging when relying solely on reference genomes.

Advances in long‐read sequencing platforms, including PacBio HiFi and Oxford Nanopore Technologies (ONT), combined with Hi‐C scaffolding, now permit chromosome‐level assemblies capable of resolving repetitive regions and complex BGCs. These improvements have substantially enhanced structural genome resolution; however, functional interpretation remains challenging because many biosynthetic loci lack experimental validation and regulatory interactions often extend beyond sequence information alone [8]. Complementing single‐genome approaches, pangenomics has emerged as a powerful framework for capturing intraspecific variation, distinguishing core from dispensable genomic components, and identifying presence/absence polymorphisms and rare alleles that contribute to chemotypic diversity [9].

Such analyses reveal that key metabolic genes often reside in the accessory genome, emphasizing the evolutionary fluidity of specialized metabolism.

Although integration of genomic, transcriptomic, proteomic, metabolomic, and epigenomic datasets has considerably improved pathway inference, translating these datasets into experimentally supported regulatory models remains a major challenge because different omics layers frequently capture distinct biological processes. The incorporation of artificial intelligence (AI) and machine‐learning models further enhances the ability to predict biosynthetic pathways, regulatory elements, and genotype‐to‐chemotype relationships. Concurrently, genome editing tools such as CRISPR/Cas systems provide an experimental framework for validating candidate genes and engineering metabolic pathways with precision. Combined, these approaches may improve the identification of useful genetic variation, support molecular breeding, facilitate conservation genomics, and contribute to the sustainable development of phytopharmaceutical resources.

Here, we emphasize systems‐level interpretation and synthesize how structural genomic features such as WGD, TEs, gene clustering, and haplotype diversity can influence the organization, expression, and regulation of biosynthetic pathways. Furthermore, we evaluate how pangenomics, QTL/GWAS mapping, and functional genomics provide evidence for potential relationships between genomic features and observable chemotypes, while recognizing that the strength of support differs among systems.

Within the scope of the present review, mechanistic genomics refers to the systems‐level interpretation of how structural genomic variation influences the organization, regulation, and expression of specialized metabolism pathways. Structural features such as transposable‐element (TE) insertions, copy‐number variation (CNV), WGD, and biosynthetic gene clusters are considered as potential contributors to metabolic phenotypes. However, many proposed genome‐to‐metabolite relationships remain supported primarily by comparative genomics and multiomics evidence. Direct functional validation is still lacking for many of these relationships.

To distinguish experimentally validated mechanisms from predictive or correlative associations, this review adopts a graded evidence framework across MAP systems.

Mechanistic evidence is categorized as follows: (1) strong evidence, supported by direct functional validation (CRISPR, transgenics, biochemical assays, metabolic flux, and enzyme characterization); (2) moderate evidence, supported by integrated multiomics supported by independent genetic evidence (QTL/GWAS) but lacking direct functional validation; and (3) preliminary evidence, based primarily on comparative genomics, coexpression, transcriptomics, metabolomics, AI prediction, computational inference, and comparative analyses. This classification enables distinguishing robust causal mechanisms from emerging hypotheses and highlights priority areas requiring experimental validation. Throughout this review, conclusions are interpreted according to this evidence framework to distinguish experimentally validated mechanisms from biologically plausible functional hypotheses.

## 2. Mechanistic Insights From Genome Sequencing of MAPs

Although chromosome‐scale assemblies have transformed MAP genomics, genome continuity alone cannot fully explain specialized metabolism because functional annotation, regulatory interactions, and metabolic validation remain incomplete for many species. Over the past decade, improvements in long‐read sequencing and scaffolding technologies have shifted MAP genomics from fragmented draft assemblies to chromosome‐scale, haplotype‐resolved genomes [3, 4]. This transition has improved understanding of how genome architecture, including repeat landscapes, duplication history, and chromosomal organization of BGCs, may contribute to phytochemical pathway evolution.

The transition from short‐read sequencing to long‐read platforms such as PacBio HiFi and ONT, together with Hi‐C chromatin scaffolding and Bionano optical mapping, has transformed MAP genomics. These technologies resolve highly repetitive and structurally dynamic genomic regions where many specialized‐metabolism loci are located [6, 10]. BGCs in MAPs are frequently enriched with tandem duplications, TEs, paralogous enzyme families, and large intergenic regulatory regions that are difficult to assemble accurately using short‐read sequencing alone. Short‐read assemblies often collapse repetitive synthase genes, fragment BGC boundaries, or misassign duplicated pathway copies, thereby obscuring the true structural organization of metabolically important loci. Long‐read sequencing has substantially improved the identification of BGCs and structural variation, thereby facilitating functional investigation of specialized metabolism.

For example, the chromosome‐scale genome of *Helianthus tuberosus* (Table 1 **)** illustrates how high‐contiguity assemblies clarify polyploid genome dynamics and illuminate duplicated metabolic pathways linked to tuber development and stress response [11, 26]. Such insights highlight how polyploidy may expand enzymatic repertoires that are proposed to support specialized metabolism.

**Table 1:** Chromosome‐scale genome assemblies of medicinal and aromatic plants (MAPs), illustrating how long‐read and chromosome‐conformation technologies resolve repetitive biosynthetic architectures and functionally informative metabolic loci.

Plant species	Genome size (Gb)	Assembly strategy	Functional insights into specialized metabolism/genome architecture	Evidence strength	References
*Cannabis sativa*	0.82	PacBio HiFi + Hi‐C	Resolved tandemly repeated cannabinoid synthase loci; revealed TE‐mediated structural variation associated with chemotype diversity	Moderate (structural genomics + chemotype association + partial functional validation)	[11, 12]
*Papaver somniferum*	2.72	ONT + Hi‐C	Defined the organization and evolutionary history of morphinan alkaloid BGCs; revealed gene duplication and neofunctionalization events including the STORR fusion gene	Strong (genetic, biochemical, and functional validation available)	[13, 14]
*Artemisia annua*	1.74	PacBio CLR + Illumina + Hi‐C	Clarified the architecture of the artemisinin pathway; identified transcriptional regulators and chromatin features linked to trichome‐specific expression	Strong (multiomics + functional pathway characterization)	[15, 16]
*Panax ginseng*	3.12	PacBio + Hi‐C	Illuminated WGD‐driven expansion of terpene synthases and glycosyltransferases associated with ginsenoside diversity; uncovered subgenome structural asymmetry	Preliminary–Moderate (comparative genomics + metabolomic association)	[17]
*Helianthus tuberosus*	21.0 (hexaploid)	PacBio HiFi + Hi‐C	Demonstrated polyploid genome reorganization; resolved duplicated pathways associated with tuber development and metabolic plasticity	Preliminary–Moderate (genome‐scale structural inference with limited metabolic validation)	[11]
*Salvia miltiorrhiza*	0.64	PacBio + Illumina + Hi‐C	Resolved diterpenoid biosynthetic gene organization; provided mechanistic insight into tanshinone pathway regulation and enzyme modules	Moderate (pathway characterization supported by transcriptomics/proteomics)	[18, 19]
*Rauvolfia serpentina*	~2.6	Hybrid Illumina + population genomics approaches	Revealed population genomic structuring and allelic diversity influencing indole alkaloid biosynthesis; provided conservation‐relevant genomic insights	Preliminary–Moderate (population genomics and association‐based evidence)	[20–22]
*Curcuma longa*	~1.56	Hybrid assembly strategies	Clarified genome organization linked to curcuminoid metabolism; identified structural and regulatory features associated with phenolic specialization	Preliminary (comparative and regulatory inference)	[21, 23]
*Ocimum basilicum*	2.13	Nanopore + Illumina (hybrid)	Revealed allelic and structural variation in terpenoid biosynthesis; identified expanded CYP450 families associated with essential‐oil differentiation	Preliminary–Moderate (comparative + metabolite association)	[3]
*Dendrobium officinale*	~1.35	PacBio + Hi‐C	Revealed expansion of polysaccharide‐ and alkaloid‐associated gene families; TE‐rich genomic regions associated with metabolic diversification and medicinal polysaccharide biosynthesis	Preliminary (comparative genomics + transcriptomic association)	[24]
*Citrus* spp.	~0.37	PacBio + Hi‐C	Identified terpene synthase diversification and structural variation in flavonoid and volatile‐metabolite biosynthetic loci associated with aromatic and medicinal compound diversity	Preliminary (comparative genomics + metabolomic association)	[25]

For example, in *Cannabis sativa*, long‐read assemblies resolved highly repetitive cannabinoid synthase regions that were previously collapsed in short‐read genomes, revealing tandem organization, CNV, and TE‐associated rearrangements that may contribute to chemotype differentiation. Similarly, in *Papaver somniferum*, long‐read sequencing enabled reconstruction of the morphinan alkaloid BGC and identification of the STORR fusion gene within structurally complex duplicated regions. These examples illustrate how improved genome continuity facilitates reconstruction of complex metabolic loci.

Similarly, genome assemblies from diverse medicinal plant families including Cannabaceae, Papaveraceae, Lamiaceae, Araliaceae, Zingiberaceae, Solanaceae, Orchidaceae, Rutaceae, and Apocynaceae have collectively provided insights into genomic features associated with the evolution of specialized metabolism. Across these species, a recurrent pattern emerges, where specialized metabolic pathways are frequently encoded by BGCs enriched in cytochrome P450s, terpene synthases (TPSs), and acyltransferases, often situated in repeat‐rich or structurally dynamic genomic regions. Recent comparative metabolite analyses of medicinal *Artemisia* cultivars further demonstrate that developmental stage‐dependent regulation strongly influences flavonoid accumulation, antioxidant activity, and medicinal compound composition, reinforcing the importance of integrating temporal metabolomics with genome‐enabled pathway analyses in MAP research [1].

Emerging genomic resources from understudied MAP families further broaden the evolutionary scope of genome‐enabled MAP genomics. In Orchidaceae, chromosome‐scale assemblies of *Dendrobium officinale* and related medicinal orchids [27] have revealed expansion of polysaccharide‐ and alkaloid‐associated gene families together with extensive TE proliferation, suggesting that genome structural plasticity contributes to bioactive metabolite diversification. Similarly, in Rutaceae, comparative genomics of *Citrus* species [28] have clarified TPS diversification, structural variation in flavonoid biosynthetic loci, and lineage‐specific metabolic adaptations associated with aromatic and medicinal compound production. In combination with emerging genomic resources from Lamiaceae, Apocynaceae, and Zingiberaceae, these studies suggest that genome‐to‐metabolite relationships are being investigated across an increasingly broad phylogenetic spectrum rather than being restricted to traditional model MAPs.

Concrete locus‐level discoveries further illustrate these relationships. Long‐read sequencing resolved cannabinoid synthase loci, providing the structural framework for subsequent pangenomic analyses [12]. In *Artemisia annua*, the amorpha‐4,11‐diene synthase (ADS) and *CYP71AV1* genes form the core catalytic module of the artemisinin pathway, with copy expansion and regulatory variation that may influence metabolite yield [15]. In *P. somniferum*, the discovery of the *STORR* fusion gene mechanistically explained the evolutionary innovation behind morphinan alkaloid biosynthesis [13]. Likewise, *Ocimum* species show diversification and differential expression of TPS gene families linked to lineage‐specific essential oil profiles [29].

These regions exhibit signatures of recent duplication, TE activity, or local chromatin rearrangement [12, 13]. These structural genomic features provide candidate regulatory loci for specialized metabolism. Their regulatory consequences are discussed in Section 4, where multiomics integration is used to explain how structural variation influences gene expression, enzyme abundance, and metabolic flux.

Evidence from comparative genomic and transcriptomic studies indicates that CNV at cannabinoid synthase loci is associated with altered cannabinoid accumulation, although the precise regulatory mechanisms remain under investigation [12, 30]. In *P. somniferum*, available evidence suggests that morphinan pathway evolution has been shaped by tandem duplication and neofunctionalization of BIA pathway genes, with structural rearrangements establishing BGC‐like configurations [13]. In *A. annua*, available evidence suggests that chromatin accessibility contributes to trichome‐specific activation of artemisinin biosynthesis. Although the relative contribution of chromatin remodeling versus transcription‐factor regulation remains under investigation, additional evidence is needed to determine how broadly these regulatory mechanisms operate across genetically diverse *Artemisia* accessions [15]. Taken together, these findings establish the structural genomic foundation for subsequent analyses of population variation, regulatory coordination, and functional validation discussed in the following sections.

Comparative analysis across *Cannabis*, *Papaver*, *Artemisia*, *Ocimum*, *Panax*, *Dendrobium*, and *Citrus* reveals that similar genomic mechanisms repeatedly contribute to phytochemical diversification despite the independent evolutionary origins of their metabolic pathways. Tandem duplication frequently expands enzyme families and may increase biosynthetic capacity, whereas TE‐associated rearrangements are proposed to reshape local regulatory architecture and influence pathway expression. However, the relative contribution of these mechanisms differs among lineages. For example, gene fusion has been convincingly demonstrated only for the STORR locus in *Papaver*, whereas CNV is most clearly associated with cannabinoid chemotype diversification in *Cannabis*. In *Panax*, WGD has played a larger evolutionary role than localized tandem duplication, whereas Orchidaceae and *Citrus* appear to rely more extensively on lineage‐specific gene‐family expansion and regulatory diversification. These comparisons indicate that common genomic principles operate across MAPs, but their evolutionary implementation remains lineage dependent and requires broader functional validation beyond a limited number of experimentally tractable species.

In parallel, TE‐mediated rearrangements contribute to local chromatin remodeling, CNV, and regulatory diversification across multiple MAP BGCs, including cannabinoid and artemisinin‐associated loci. Collectively, these processes indicate that MAP metabolic evolution frequently proceeds through iterative cycles of duplication, structural rearrangement, regulatory rewiring, and neofunctionalization, although the relative evolutionary rates and stability of these mechanisms likely differ among metabolite classes and genome architectures.

Among currently available MAP systems, *Papaver* provides one of the strongest experimentally validated examples of a mechanistic genome‐to‐metabolite relationship through biochemical and genetic characterization of STORR.

### 2.1. Common Functional Principles Emerging Across MAP Genomes

Although specialized metabolic pathways have evolved independently across (MAP) lineages, comparative genomic studies have identified recurrent genomic features associated with phytochemical diversification. Rather than acting independently, structural genomic features may influence different layers of molecular regulation, contributing to variation in metabolic pathways and specialized‐metabolite accumulation. This systems‐level hierarchy provides a unifying framework that explains how genome architecture is translated into chemotype diversity across diverse MAP species (Table 2).

**Table 2:** Conceptual summary of proposed genomic mechanisms associated with specialized‐metabolite diversification in medicinal and aromatic plants (MAPs).

Genome feature	Primary molecular mechanism	Metabolic consequence
Tandem duplication	Increased enzyme dosage and functional divergence	Increased pathway flux and metabolite accumulation
Transposable‐element (TE) insertion	Creation of novel cis‐regulatory elements and transcription‐factor binding sites	Altered pathway expression and metabolic plasticity
Copy‐number variation (CNV)	Gene dosage imbalance and differential transcription	Chemotype differentiation
Whole‐genome duplication (WGD)	Gene neo‐functionalization and sub‐functionalization	Emergence of novel metabolic pathways and metabolites
Chromatin accessibility	Coordinated activation of biosynthetic gene clusters (BGCs)	Tissue‐specific and environmentally responsive biosynthesis

Tandem duplication is one of the most prevalent mechanisms underlying the expansion of specialized metabolism genes in MAPs. Duplication of TPSs, cytochrome P450 monooxygenases, methyltransferases, and glycosyltransferases increases gene dosage. However, duplicated genes often remain functionally redundant or undergo pseudogenization, indicating that duplication alone is insufficient to generate metabolic innovation.

One duplicated copy often retains the ancestral catalytic activity, whereas additional copies accumulate sequence variation that broadens substrate specificity or acquires novel biochemical functions. This process may expand pathway capacity and may redirect metabolic flux toward the production of lineage‐specific specialized metabolites. Examples include the expansion of cannabinoid synthase genes in *C. sativa*, TPS families in *Ocimum basilicum*, and glycosyltransferase families involved in ginsenoside biosynthesis in *Panax ginseng*, where tandem duplication is proposed to contribute to increased phytochemical complexity.

TE insertions may contribute to metabolic diversification by reshaping the regulatory landscape of biosynthetic loci rather than simply increasing genomic variability. TE‐derived sequences frequently introduce novel promoters, enhancers, and transcription‐factor binding motifs, while also modifying local chromatin organization and epigenetic states. These regulatory alterations influence the spatial, temporal, and environmental responsiveness of biosynthetic gene expression. In several MAPs, TE‐rich genomic regions are enriched around BGCs, suggesting that repeated TE‐mediated regulatory rewiring may facilitate adaptive modulation of specialized metabolism. However, distinguishing adaptive regulatory rewiring from neutral TE insertion remains difficult in many MAP genomes because direct functional evidence is still limited.

CNV provides another important mechanism linking genome architecture to metabolic phenotype. Alterations in the number of biosynthetic gene copies may modify enzyme dosage and consequently influence pathway throughput and metabolite accumulation. CNV at cannabinoid synthase loci is consistently associated with chemotype variation; however, current evidence suggests that chromatin regulation, promoter variation, and tissue‐specific expression also contribute substantially, and causal relationships remain incompletely resolved. Examples of CNV in MAPs, including *Cannabis*, are discussed in Section 2. Here, CNV is considered as a general functional principle whereby gene dosage may influence specialized metabolism, although its contribution varies among species and regulatory contexts.

WGD is widely considered an important evolutionary driver of metabolic innovation throughout plant evolution. Polyploidization generates duplicated biosynthetic genes that subsequently undergo neofunctionalization, subfunctionalization, or differential regulatory specialization. These evolutionary processes may increase biochemical complexity by allowing duplicated enzymes to catalyze new reactions, function in different tissues, or respond to distinct developmental or environmental signals. Consequently, WGD expands both the catalytic repertoire and regulatory flexibility of specialized metabolism networks. In MAPs such as *P. ginseng*, duplicated TPSs and UDP‐glycosyltransferases have diversified following genome duplication, contributing to the structural complexity and diversity of ginsenosides. Although WGD expands evolutionary potential, only a subset of duplicated genes is retained and subsequently recruited into specialized metabolism.

Regulatory architecture provides the regulatory interface through which structural genomic variation is translated into coordinated pathway activity. BGCs frequently require synchronized activation of multiple genes, a process that depends on local chromatin organization, histone modifications, DNA methylation, and transcription‐factor recruitment. Accessible chromatin is associated with coordinated transcription of pathway enzymes, whereas condensed chromatin restricts biosynthetic activity to specific tissues or developmental stages. This regulatory mechanism is particularly evident in glandular trichomes and other specialized secretory tissues, where localized chromatin remodeling enables coordinated activation of biosynthetic pathways. Dynamic chromatin regulation is proposed to link genome architecture with tissue‐specific metabolism and environmentally responsive phytochemical production.

Taken together, comparative evidence across diverse MAP families indicates that structural genomic variation consistently influences specialized metabolism through a limited set of recurrent mechanisms, including tandem duplication, TE activity, CNV, WGD, and chromatin remodeling. Nevertheless, the evolutionary importance of each mechanism varies substantially among lineages depending on genome organization, metabolite class, and ecological adaptation. This comparison highlights that no single genomic mechanism universally explains phytochemical diversification, reinforcing the need for lineage‐specific functional studies. This framework provides the conceptual basis for the following sections, which discuss complementary approaches for investigating genome‐to‐metabolite relationships.

Yet despite these advances, genome assemblies alone capture only part of the biological complexity. Many MAP genomes remain incomplete or poorly annotated, particularly those with large genome sizes or extreme repeat content [3]. Functional annotation of metabolic genes remains uneven, and BGC boundaries are often ambiguous due to paralogy, pseudogenization, or tissue‐specific gene expression. Despite recent progress, currently available chromosome‐scale assemblies remain disproportionately concentrated in economically important species such as *Cannabis*, *Papaver*, *Artemisia*, and *Panax*. Consequently, many medicinally important families including Orchidaceae, Rutaceae, Apocynaceae, and Zingiberaceae remain comparatively underrepresented, limiting the generalization of genome‐enabled genomic models across MAP diversity.

**Figure 1:**
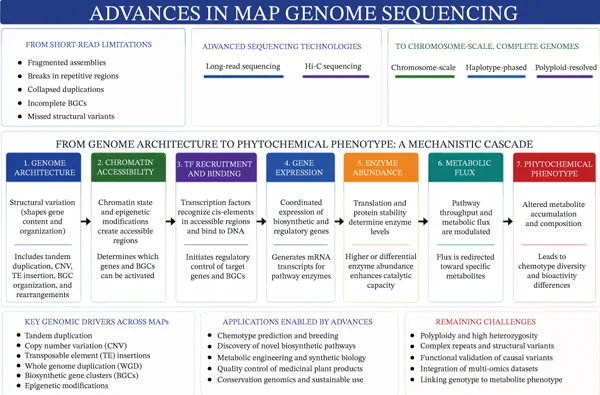
Conceptual overview of chromosome‐scale genome sequencing and long‐read technologies highlighting structural genomic features, including tandem duplication, copy‐number variation (CNV), transposable‐element (TE) insertions, and biosynthetic gene clusters (BGCs), associated with phytochemical diversity in medicinal and aromatic plants (MAPs). The figure is intended as a conceptual illustration only. (Created using https://www.biorender.com).

A second challenge is that single reference genomes do not adequately represent intraspecies metabolic diversity. Structural variants, presence/absence polymorphisms, and allelic differences can strongly influence chemotype, yet these features remain largely invisible without multiaccession sequencing. As summarized in (Table 1) and conceptually illustrated in (Figure 1), reference‐quality assemblies provide a framework for evolutionary and functional analyses, but they must be complemented by pangenomic frameworks to uncover the full range of genetic variation that contributes to phytochemical traits. This is particularly critical for MAPs, where specialized metabolism evolves rapidly and may be distributed across dispensable genomic regions.

Although chromosome‐scale genome assemblies have substantially advanced MAP genomics, comprehensive interpretation of phytochemical diversity requires complementary genomic approaches. These are discussed in the following section.

All schematic figures presented in this review are intended as conceptual summaries of published evidence rather than quantitative meta‐analyses. Visual properties such as color intensity, icon size, and spatial arrangement are used only for graphical clarity and do not represent statistical weighting or measured biological effects.

### 2.2. Why MAPs Require a Distinct Functional Genomic Framework

MAPs differ fundamentally from conventional crop species because their evolutionary success and biological value are largely shaped by chemically diverse specialized metabolites rather than traits associated primarily with yield, domestication, or biomass improvement. Unlike major crops, where breeding has frequently targeted relatively narrow agronomic traits, MAP evolution has been strongly influenced by ecological interactions involving defense, pollinator attraction, environmental adaptation, and chemical diversification.

This section highlights the biological characteristics that distinguish MAP genomes from those of conventional crop species. Unlike primary metabolic genes, specialized‐metabolism genes frequently occur within evolutionarily dynamic genomic regions shaped by duplication, rearrangement, TE activity, and biosynthetic gene clustering. Importantly, plant BGCs differ substantially from microbial operon‐like BGCs. [31] Microbial clusters are usually compact, cotranscribed units optimized for coordinated pathway expression, whereas plant BGCs are more loosely organized, embedded within chromatin‐regulated genomes, and controlled by tissue‐specific, developmental, and environmental regulatory networks.

Another defining characteristic of MAPs is that many specialized metabolites are synthesized within highly specialized anatomical structures, including glandular trichomes, resin ducts, secretory cavities, and laticifers, where genome regulation is spatially coordinated with metabolite production. These secretory structures require precise coordination between genome architecture and regulatory control, including chromatin accessibility, transcription‐factor recruitment, enhancer activity, and epigenetic regulation that restrict biosynthetic pathway activation to specific cell types. Therefore, MAP chemotypes often reflect not only the presence of biosynthetic genes but also when, where, and how these genomic regions are transcriptionally activated.

Consequently, transcript abundance alone is often insufficient to explain metabolite accumulation, necessitating the integration of genomics with epigenomics, transcriptomics, proteomics, and metabolomics to establish causal genome‐to‐metabolite relationships. Therefore, a MAP‐specific systems‐level genomic framework must integrate genome architecture, evolutionary history, spatial regulation, and specialized cellular contexts rather than simply cataloging biosynthetic genes. However, the relative contribution of these mechanisms differs among MAP lineages, and direct experimental validation remains necessary to distinguish causal regulatory mechanisms from genome‐level associations.

## 3. Pangenomics in MAPs

Pangenomics provides a powerful framework [32]; however, functional interpretation increasingly depends on integrating structural variation with functional genomic evidence. Although pangenomic analyses have substantially improved identification of structural variation associated with phytochemical diversity, relatively few candidate variants have been experimentally validated. Accordingly, structural variants identified through pangenomics provide candidate loci for further investigation, consistent with the evidence framework introduced earlier. Structural variants become biologically meaningful only when they modify regulatory networks controlling specialized metabolism. PAV and CNV primarily define structural diversity among accessions [9, 33]. Their downstream regulatory consequences are evaluated through multiomics integration in Section 4.

Unlike many agronomic traits that are predominantly controlled by conserved core genes, specialized metabolism in MAPs is frequently associated with structurally dynamic regions of the accessory genome. BGCs, duplicated synthases, regulatory transcription factors, and transporter genes are often absent from the reference genome or vary substantially among accessions. Consequently, pangenomic analyses are particularly valuable in MAPs because they capture the genomic diversity directly responsible for chemotype differentiation rather than merely expanding catalogs of structural variation.

Together, these observations indicate that pangenomic variation may contribute to specialized metabolism by altering both gene content and regulatory architecture [34].

Thus, pangenomic structural variation represents a potential contributor to regulatory variation rather than merely a source of sequence diversity, because it may influence pathway coordination and specialized‐metabolite production depending on genomic context.

These forms of structural variability are biologically important because specialized metabolic pathways especially those encoded by (BGCs) frequently occupy genomic regions characterized by duplication, recombination, or local chromatin remodeling [9].

Presence‐absence variation (PAV) and CNV within BGCs can alter specialized metabolism through several interconnected molecular processes. CNV frequently may change enzyme dosage by increasing or reducing the number of active biosynthetic gene copies, thereby modifying pathway flux and downstream metabolite accumulation. Similarly, PAV may result in the gain or loss of pathway enzymes, transporters, or transcriptional regulators required for metabolite biosynthesis, resulting in accession‐specific chemotypes. Structural variation within or surrounding BGCs may additionally influence promoter architecture, chromatin accessibility, transcription‐factor recruitment, and coordinated expression of clustered pathway genes. As a result, pangenomic variation may affect not only gene content but also regulatory synchronization and metabolic efficiency across specialized‐metabolism pathways.

For MAPs, pangenomes have been particularly revealing in showing how genome architecture underlies chemotype diversity. Although most existing pangenome efforts have focused on crops, emerging datasets across *Cannabis, Ocimum, Panax, Capsicum, Mentha* [35], *Withania*, and additional medicinal families suggest that metabolite‐associated PAV and CNV are widespread across phylogenetically diverse MAPs rather than being restricted to a few model systems. Traditional reference genomes cannot capture the allelic richness that differentiates metabolite profiles across cultivars, landraces, or wild populations. In contrast, pangenomic analyses identify accession‐specific BGC compositions, gene family expansions, and regulatory elements that modulate the biosynthesis of essential oils, alkaloids, terpenoids, and other specialized metabolites.

The structural and functional significance of species (e.g., *Cannabis*, *Papaver*, *Artemisia*, and *Panax*) has already been discussed in Section 2. Here, these examples are revisited only to illustrate how pangenomic analyses capture accession‐level genomic diversity rather than to repeat species‐specific biological mechanisms [12, 13]. These studies, summarized in (Table 3 **)**, illustrate how pangenomic variation provides insights into possible mechanisms underlying lineage‐specific metabolic diversity.

**Table 3:** Functional insights from emerging pangenomic studies in medicinal and aromatic plants.

Plant species	Pangenome/comparative approach	Key functional insights into specialized metabolism	Primary evidence supporting the mechanism	Validation level	References
*Cannabis sativa*	Multiaccession comparative genome	CNV may alter synthase dosage and pathway flux underlying chemotype differentiation	Structural genomics + chemotype association + partial functional validation	Moderate	[12]
*Papaver somniferum*	Multigenotype genome comparison	PAV may modify coordinated pathway expression and alkaloid biosynthetic capacity	Comparative genomics + transcriptomics + biochemical characterization of STORR pathway	Moderate	[3]
*Ocimum basilicum*	Intraspecific comparative genomics	Allelic and structural diversification in terpenoid biosynthetic genes is associated with essential‐oil variation	Comparative genomics + transcriptomics + metabolomic profiling	Moderate	[36]
*Panax ginseng*	Multiaccession resequencing	WGD‐driven expansion and CNV in ginsenoside pathway genes correlate with metabolite diversity	Comparative genomics + metabolomics + population genomics	Preliminary‐Moderate	[17]
*Capsicum spp.*	Species‐level pangenome	Dispensable genome contains capsaicinoid pathway genes and stress‐responsive loci	Pangenomics + GWAS + metabolite association	Moderate	[37]
Cross‐species MAP families	Super‐pangenome analyses	Conserved pathway cores and lineage‐specific BGC expansions are proposed to contribute to metabolic diversification	Comparative evolutionary genomics + computational inference	Preliminary	[9]

From a regulatory perspective, structural variation surrounding benzylisoquinoline alkaloid biosynthetic genes modifies coordinated transcription of pathway enzymes and changes metabolic flux through the morphinan branch, ultimately generating accession‐specific alkaloid profiles.

Across MAPs, pangenomic analyses consistently demonstrate that accession‐specific structural variation modifies biosynthetic gene content and regulatory architecture. The specific biological functions of loci have been discussed earlier (Section 2); here, emphasis is placed on population‐level genomic diversity rather than individual pathway mechanisms.

Beyond individual model species, comparative pangenomic analyses are increasingly extending across medicinal plant families, enabling evaluation of whether structural genomic mechanisms identified in *Cannabis* or *Papaver* represent conserved or lineage‐specific evolutionary patterns. Building on the *Cannabis* example discussed in Section 2, pangenome analyses further demonstrate how accession‐level CNV and dispensable‐genome variation differ among germplasm collections [12]. These comparative datasets illustrate that structural genomic diversity extends beyond single‐reference assemblies and differs substantially among populations and lineages.

These comparative analyses reveal both conserved metabolic cores and lineage‐specific BGC expansions, clarifying how duplication, sub functionalization, and regulatory rewiring have repeatedly generated novel chemical scaffolds across evolutionary time [9]. For instance, in *Capsicum* species, pangenome analyses show that the dispensable genome encodes key genes for capsaicinoid biosynthesis, highlighting how structural variation outside the core genome can drive ecological adaptation and specialized metabolic phenotypes [37]. Functionally, structural variation within the dispensable genome changes the dosage and regulation of capsaicinoid biosynthetic genes, thereby influencing pathway activity, capsaicinoid accumulation, and pungency phenotypes.

Collectively, these studies suggest that pangenomes consistently identify accessory‐genome variation associated with specialized metabolism, but the nature of that variation differs among lineages. Alkaloid‐producing species often exhibit diversification through structural rearrangements and biosynthetic gene‐cluster evolution, whereas terpene‐producing species more commonly show expansion of TPS families and regulatory variation. These lineage‐specific patterns indicate that pangenomics reveals both conserved principles of genome evolution and unique evolutionary trajectories that reflect distinct biosynthetic strategies.

These dispensable genomic regions frequently harbor regulatory elements, duplicated biosynthetic genes, and TE‐associated chromatin modifications that alter transcriptional coordination of specialized‐metabolism pathways. Consequently, pangenomic variation may influence not only gene presence but also regulatory architecture controlling metabolite biosynthesis, pathway responsiveness, and chemotype stability across accessions. Similar comparative genomic trends are emerging in Orchidaceae and Rutaceae, where structural diversification of TPS, flavonoid, and alkaloid‐associated loci appears to contribute to lineage‐specific phytochemical variation. Although pangenomic resources remain comparatively limited for Orchidaceae, Rutaceae, Apocynaceae, and Zingiberaceae, their increasing availability is substantially improving the taxonomic breadth of MAP genomics and reducing historical dependence on a small number of model medicinal species.

**Figure 2:**
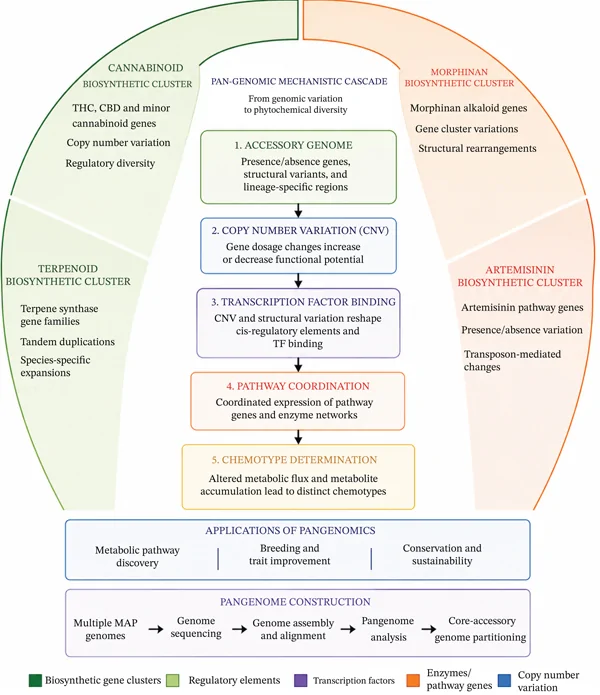
Conceptual overview of how pangenomic variation contributes to specialized‐metabolite diversity in medicinal and aromatic plants (MAPs). The figure summarizes published concepts and is intended as a conceptual illustration only. (Created using https://www.biorender.com).

These relationships are conceptually summarized in (Figure 2), illustrating how pangenomics may help interpret connections between accession‐specific genomic variation and specialized metabolism. Structural variants may influence specialized metabolism by modifying regulatory architecture, including transcription‐factor recruitment, chromatin accessibility, enzyme dosage, and coordinated activation of BGCs. Accordingly, pangenomic analyses establish a genome‐enabled bridge linking genome architecture with metabolic pathway organization and chemotype differentiation rather than simply cataloging genomic polymorphism. This perspective reframes pangenomics as more than a tool for cataloging variation: it becomes a mechanistic bridge linking genome architecture, evolutionary processes, and metabolic output. As highlighted by recent plant pan‐genomic frameworks, graph‐based and super‐pangenome approaches are increasingly recognized as essential for resolving structurally complex genomic regions, including (BGCs), repetitive loci, and dispensable metabolic genes that are often missed in linear reference assemblies [34].

Future progress in MAP pangenomics will depend on integrating structural variation with complementary multiomics and functional genomic datasets. Experimental validation will remain essential for confirming genome‐to‐metabolite relationships and distinguishing causal variants from neutral polymorphisms.

### 3.1. Functional Convergence Across MAP Pangenomes

Despite considerable taxonomic diversity among MAPs, comparative pangenomic studies reveal a conserved regulatory framework. Structural variants including PAV, CNV, tandem duplication, and TE‐mediated rearrangements recurrently influence enzyme dosage, cis‐regulatory architecture, chromatin accessibility, and transcriptional coordination of BGCs. These molecular effects may alter pathway flux and metabolic efficiency, ultimately producing lineage‐specific phytochemical profiles. Thus, pangenomics provides a systems‐level explanation for chemotype diversification by identifying not only which genomic regions vary among accessions, but also how those structural differences modify regulatory networks governing specialized metabolism.

### 3.2. Genotype‐Phenotype Association Frameworks Linking Genomic Variation With Metabolite Traits

Although pangenomics identifies structural and accession‐specific genomic variation, genotype‐phenotype association frameworks such as QTL mapping, GWAS, and haplotype analysis are required to functionally connect this variation with metabolite phenotypes and chemotype diversity. These approaches therefore provide statistical frameworks for investigating associations between genome architecture and specialized metabolism traits rather than as direct components of pangenomic analysis itself.

In MAP pangenomic and association studies, reproducibility increasingly depends on standardized analytical thresholds and population‐scale sampling strategies. Most GWAS analyses linking genomic variation with metabolite traits typically employ population sizes ranging 100–500 accessions, minor allele frequency (MAF) filters of > 0.05, and genome‐wide significance thresholds between 10^−5^ and 10^−8^ depending on marker density and population structure. Similarly, QTL mapping studies commonly use logarithm of odds (LOD) thresholds > 3.0 for significant metabolite‐associated loci. For structural‐variation analyses, CNV and PAV are generally validated through long‐read assemblies, read‐depth normalization, and transcript‐supported confirmation to reduce false‐positive metabolic associations.

In several MAP systems, QTL and GWAS studies have suggested that variation in specialized metabolites is frequently governed by structural variation, copy number diversity, or presence/absence of biosynthetic genes rather than simple SNP variation. For example, in *Capsicum* species, GWAS and population resequencing revealed that key capsaicinoid biosynthesis genes reside in structurally dynamic genomic regions, where presence‐absence polymorphisms and regulatory variation underlie pungency and metabolite composition [37]. These results demonstrate how dispensable‐genome content and local structural remodeling directly translate to metabolite phenotype, complementing pangenomic inferences.

Extending the *Cannabis* example introduced in Section 2, haplotype‐resolved analyses demonstrate how structural genomic variation can be linked with genotype‐phenotype association studies [12]. These findings illustrate how haplotype structure, rather than gene presence alone, governs metabolic outcomes, reinforcing the value of haplotype‐resolved genomics in MAP research.

Comparable genetic linkage principles apply to alkaloid‐producing MAPs. In *P. ginseng*, population genomic datasets have identified CNV and allelic diversification within TPS and glycosyltransferase families associated with differences in ginsenoside composition [17]. Such cases underscore how QTL and haplotype analyses clarify which duplicated or variant pathway copies are functionally consequential for metabolite output, although additional validation through functional genomics remains necessary before assigning causal biological roles.

Taken together, QTL, GWAS, and haplotype‐resolved analyses validate and refine pangenomic discoveries by (1) identifying metabolite‐associated loci and haploblocks, (2) distinguishing neutral structural variation from functionally relevant alleles, and (3) linking metabolic diversity to specific evolutionary processes such as duplication, TE activation, or subgenome differentiation. Importantly, these tools establish direct genotype‐to‐chemotype relationships, enabling predictive breeding, precision genome editing, and targeted pathway engineering. GWAS and QTL mapping have identified numerous metabolite‐associated loci; not all reported associations have been consistently replicated across independent populations. Population structure, environmental variation, and experimental design may contribute to these discrepancies, emphasizing that association signals require functional validation before causal biological roles can be assigned.

In this framework, pangenomics defines the landscape of structural genomic diversity, whereas genotype‐phenotype association analyses identify which variants exert functional effects on metabolic traits. Thus, although pangenomics reveals structural diversity, QTL and GWAS identify statistically associated loci that provide candidates for subsequent mechanistic investigation.

Nevertheless, many metabolite‐association studies remain constrained by limited population size, environmental replication, and insufficient validation across independent accessions or cultivation conditions. Future GWAS and QTL analyses should incorporate replicated multienvironment datasets and experimentally validated candidate loci to reduce false‐positive genotype‐to‐chemotype associations.

GWAS and QTL analyses strengthen genotype‐to‐chemotype inference by identifying candidate loci for functional validation. However, distinguishing causal structural variants from neutral genomic polymorphisms remains a major challenge requiring complementary functional genomics.

## 4. Multiomics Integration for Pathway Discovery

Multiomics integration combining genomics, transcriptomics, proteomics, metabolomics, and epigenomics enables a systems‐level reconstruction of how metabolic pathways are organized, regulated, and executed in planta [38–40], although successful integration remains dependent on consistent experimental design, tissue sampling, and analytical reproducibility. Multiomics integration enables inference of regulatory networks and prioritization of candidate regulatory interactions, although many inferred relationships remain to be experimentally validated. This approach is particularly powerful for MAPs, where specialized metabolism depends on complex regulatory hierarchies, tissue‐specific biosynthesis, and extensive gene family diversification [9]. By linking structural variation with expression dynamics and metabolite accumulation, multiomics can provide the mechanistic resolution needed to identify (BGCs), regulatory modules, and metabolic flux control points.

Rather than revisiting species‐specific genomic discoveries described in previous sections, this section emphasizes how multiomics integration functionally interprets those genomic observations across diverse MAP lineages.

Importantly, multiomics integration enables mechanistic reconstruction of the intermediate regulatory layers connecting genome architecture with metabolite phenotype. For example, structural variation or duplication identified genomically can be linked to altered chromatin accessibility, transcription‐factor activity, enzyme abundance, and ultimately metabolite accumulation profiles. This systems‐level framework helps generate testable models of pathway regulation and metabolic specialization.

Genomics‐transcriptomics integration provides the first mechanistic layer, connecting gene structure with expression patterns. Although genomic assemblies and pangenomes define candidate biosynthetic genes, transcriptomics reveals the spatial and temporal specificity of their activation. Coexpression analyses across tissues or developmental stages can pinpoint pathway modules and transcription factors underlying metabolite production. For example, transcriptomic integration complements structural genomic analyses by identifying tissue‐specific regulatory modules associated with specialized metabolism [13, 16]. Nevertheless, transcript abundance alone frequently fails to predict enzyme activity or metabolite accumulation.

Consistent with the genomic patterns described earlier for *Ocimum*, comparative transcriptomics indicates that expression divergence among TPS alleles is associated with chemotype diversity [36]. Comparable multiomics frameworks are now being applied across *Salvia*, *Curcuma* [23], *Rauvolfia*, and *Taxus*, revealing lineage‐specific recruitment of enzymes and regulatory modules underlying diterpenoids, curcuminoids, indole alkaloids, and taxanes. Similar integrative approaches are increasingly being applied in medicinal orchids such as *D. officinale* and in Rutaceae species including *Citrus*, where transcriptomic‐metabolomic integration has revealed regulatory coordination of flavonoid, terpene, and polysaccharide biosynthesis pathways associated with medicinal quality traits. These expanding studies indicate that multiomics analyses now extend well beyond the representative species introduced in Sections 2 and 3, encompassing an increasingly diverse range of medicinal plant families.

Across these diverse MAP systems, multiomics integration consistently improves functional interpretation by linking structural genomic variation with transcriptional regulation and metabolite accumulation. However, the biological questions addressed differ among lineages. Terpenoid‐producing species primarily use multiomics to resolve enzyme‐family diversification and pathway regulation, whereas alkaloid‐producing species increasingly rely on integrated datasets to reconstruct complex biosynthetic networks. These differences illustrate how similar analytical frameworks can uncover lineage‐specific regulatory architectures despite sharing common genomic principles.

Multiomics studies can improve functional interpretation of candidate loci. Loci introduced in the genome‐sequencing section have subsequently served as model systems for multiomics integration, demonstrating how transcriptomic, proteomic, and metabolomic data improve functional interpretation of previously identified genomic features.

Proteomics and metabolomics add the functional dimension, linking enzyme abundance and activity to metabolite output. Such integrative metabolomic approaches are increasingly important for evaluating pharmacologically relevant traits in food‐medicine homologous plants, where developmental stage, environmental conditions, and genotype collectively influence antioxidant activity, flavonoid accumulation, and therapeutic metabolite composition [1]. Proteomics can resolve which gene products are synthesized and posttranslationally modified, whereas metabolomics via LC–MS/MS, GC–MS, and molecular networking quantifies pathway intermediates and downstream metabolites. In *Salvia miltiorrhiza*, proteomic and metabolomic integration uncovered candidate enzymes and regulatory proteins involved in tanshinone biosynthesis [18]. In *P. ginseng*, metabolite profiling in conjunction with coexpression analysis highlights the importance of integrating protein abundance with enzymatic activity and metabolite profiling [17].

Epigenomics provides a regulatory overlay that explains how chromatin environment shapes specialized metabolism. DNA methylation, histone modifications, and chromatin accessibility can modulate the expression of BGCs or transcriptional hubs. Multiomics studies further suggest that epigenetic regulation contributes to biosynthetic gene expression, although direct causal relationships remain experimentally unresolved across most MAP systems.[9, 30].

Epigenomic analyses in MAPs similarly require more rigorous validation frameworks. Many chromatin‐accessibility and DNA‐methylation studies rely primarily on correlative associations without independent validation of regulatory activity through reporter assays, histone‐mark enrichment analysis, or perturbation experiments. In several MAP systems, chromatin‐state inference has not been systematically compared across tissues, developmental stages, or environmental conditions, limiting mechanistic interpretation of metabolite‐associated regulatory regions. Future studies integrating ATAC‐seq, ChIP‐seq, and targeted epigenetic editing with tissue‐specific controls will be essential for validating causal regulatory mechanisms underlying specialized metabolism.

Computational frameworks integrate these diverse datasets into predictive models of pathway architecture. Network‐based coexpression analyses such as WGCNA and integrative correlation frameworks support biological interpretation of coordinated pathway regulation by identifying metabolite‐associated transcriptional modules and tissue‐specific biosynthetic networks. These tools have been successfully applied to reconstruct biosynthetic modules for alkaloids, terpenoids, phenolics, and other specialized metabolites across multiple MAP lineages [9].

Multiomics reproducibility further depends on transparent analytical criteria. Coexpression network analyses such as WGCNA generally apply Pearson correlation thresholds above 0.7–0.9 together with false‐discovery‐rate (FDR)‐corrected significance values (typically FDR < 0.05) to define metabolite‐associated gene modules. Differential‐expression analyses frequently use log_₂_ fold‐change cutoffs ≥ 1–2 combined with adjusted *p* − values < 0.05, whereas metabolomic pathway associations are commonly validated through LC‐MS/MS feature alignment, metabolite annotation confidence scoring, and replicate‐based normalization strategies.

**Figure 3:**
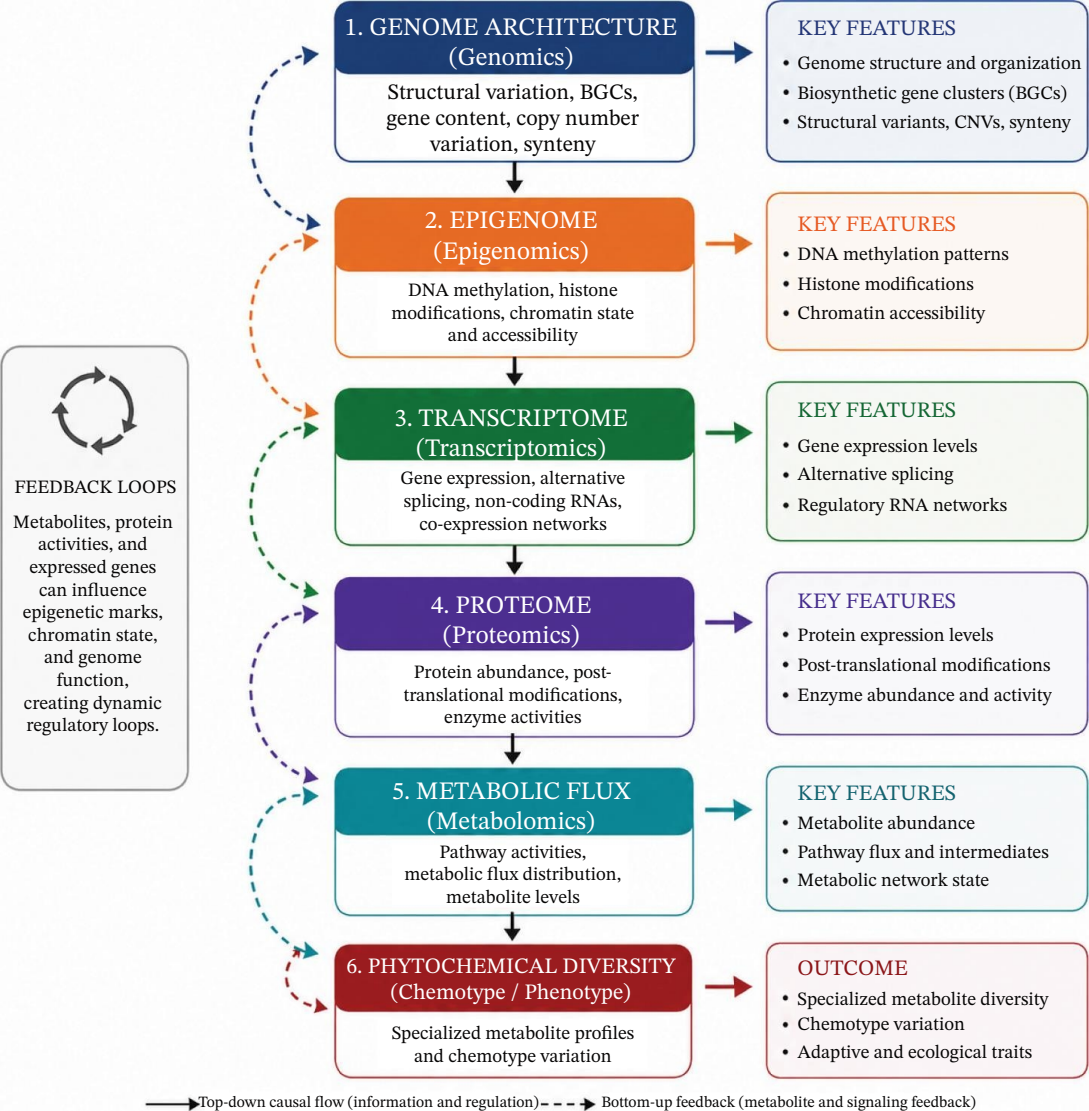
Mechanistic multiomics integration underlying specialized metabolism in medicinal and aromatic plants (MAPs). Structural genomic variation influences epigenomic regulation, transcriptomic activity, proteome dynamics, and metabolic flux, ultimately determining phytochemical composition and chemotype diversity. Feedback interactions between molecular and metabolic layers enable dynamic regulation of specialized‐metabolite biosynthesis. (Created using https://www.biorender.com).

Together these advances substantially improve pathway reconstruction; however, experimental validation remains essential before computationally inferred regulatory networks can be considered mechanistically established. (Table 4 **)** summarizes key MAP systems in which multiomics integration has uncovered pathway genes, regulatory circuits, or metabolic flux patterns. (Figure 3 **)** conceptually illustrates the multiomics discovery pipeline: genomics defines candidate loci, transcriptomics identifies active modules, proteomics and metabolomics validate enzyme function and chemical output, and epigenomics contextualizes regulatory control. Relative positions, colors, and graphical emphasis in the figure are intended for visualization only and do not represent quantitative weighting of individual omics layers. This hierarchical framework transforms static genome assemblies into dynamic biological models of specialized metabolism. The computational and predictive AI frameworks that extend these biologically interpreted multiomics networks toward automated pathway discovery and engineering applications are discussed separately in Section 6. Although multiomics integration substantially improves pathway prediction accuracy, many inferred regulatory interactions still require validation through reverse genetics, enzyme assays, or genome editing approaches.

**Table 4:** Multiomics applications in medicinal and aromatic plants (MAPs), highlighting representative evidence types supporting proposed regulatory mechanisms and their current validation status.

Plant species	Omics integrated	Key pathway/metabolite	Insight gained	Primary evidence supporting the mechanism	Type of mechanistic support	Reference
*Artemisia annua*	Genomics + transcriptomics + metabolomics	Artemisinin	Regulatory transcription factors identified for artemisinin biosynthesis	Coexpression analysis + metabolite profiling + functional characterization of pathway genes	Coexpression validated + metabolite‐correlated	[16]
*Papaver somniferum*	Genome + tissue‐specific transcriptomics	Morphinan alkaloids	Coexpression of biosynthetic genes and pathway regulation	Tissue‐specific transcriptomics + biochemical characterization of the STORR pathway	Coexpression validated; partial functional support	[13, 14]
*Salvia miltiorrhiza*	Proteomics + metabolomics	Tanshinones	Key enzymes and protein regulators characterized	Proteomics + metabolomics + enzyme characterization	Proteomics‐supported + metabolite‐correlated	[18]
*Panax ginseng*	Transcriptomics + metabolomics	Ginsenosides	Integration revealed regulatory network and metabolite flux	Coexpression network analysis + metabolomics	Association‐based multiomics inference	[17]
*Cannabis sativa*	Genome + metabolomics + epigenomics	Cannabinoids	Epigenetic regulation of cannabinoid synthase genes	DNA methylation profiling + metabolomics + comparative genomics	Epigenomics‐supported association; functional validation limited	[30]
*Ocimum basilicum*	Pangenome + transcriptomics + metabolomics	Terpenoids (essential oils)	Gene variation linked to chemotype diversity	Comparative genomics + transcriptomics + metabolite profiling	Association‐based evidence	[36]

Candidate genes identified from these studies have subsequently been investigated using CRISPR. By bridging discovery with functional engineering, these tools will accelerate the development of phytopharmaceutical platforms and enable rational manipulation of metabolic traits in MAPs.

Across MAP systems, a generalized multiomics framework is emerging in which genomics defines structural pathway architecture, transcriptomics identifies tissue‐specific regulatory activation, proteomics resolves enzyme abundance and posttranslational regulation, metabolomics quantifies pathway flux and chemical output, and epigenomics contextualizes chromatin‐state control of specialized metabolism. Integration of these datasets through coexpression analysis, network inference, and AI‐assisted modeling enables reconstruction of causal regulatory hierarchies linking genome structure with metabolite phenotype. This framework provides a transferable systems‐level model applicable across diverse metabolite classes including cannabinoids, alkaloids, terpenoids, phenylpropanoids, and triterpenoids.

The conserved mechanistic relationships linking genome architecture, regulatory mechanisms, biosynthetic pathway activity, and phytochemical phenotypes across representative MAPs are summarized in (Table 5).

**Table 5:** Proposed relationships between genome architecture and specialized‐metabolite diversity in medicinal and aromatic plants (MAPs).

Genome architectural feature	Primary regulatory mechanism	Effect on biosynthetic pathway	Resulting phytochemical phenotype	MAP species
Tandem gene duplication	Increased enzyme dosage	Enhanced pathway flux	Increased cannabinoid accumulation	*Cannabis sativa*
STORR gene fusion	Evolution of a novel catalytic enzyme	Diversion into the morphinan branch	Morphine biosynthesis	*Papaver somniferum*
Transposable‐element (TE) insertion	Promoter remodeling and regulatory rewiring	Altered pathway gene expression	Enhanced artemisinin accumulation	*Artemisia annua*
Copy‐number variation (CNV)	Gene dosage alteration	Increased terpene biosynthesis	Essential‐oil chemotype diversity	*Ocimum basilicum*
Whole‐genome duplication (WGD)	Gene neo‐/subfunctionalization	Expansion of biosynthetic pathways	Ginsenoside diversification	*Panax ginseng*

These examples demonstrate a common genome architecture‐to‐metabolite framework that provides the mechanistic foundation for the functional validation and pathway engineering strategies discussed in the following section.

(Figure 3) summarizes the mechanistic cascade reconstructed by multiomics integration, where genome architecture influences chromatin accessibility, transcription‐factor activity, enzyme abundance, pathway flux, and metabolite accumulation. This framework illustrates how multiple omics layers collectively explain causal genotype‐to‐phenotype relationships.

### 4.1. Current Limitations and Strength of Available Evidence

Although increasing numbers of chromosome‐scale genomes have accelerated mechanistic investigations of specialized metabolism, the strength of evidence varies considerably among MAP species. Many reported associations between structural genomic variation and metabolite accumulation are derived from comparative genomics, pangenome analyses, transcriptomics, or coexpression networks, whereas direct functional validation through genome editing, reverse genetics, enzyme assays, or transgenic studies remains relatively limited. Consequently, relationships involving CNV, biosynthetic gene‐cluster organization, and regulatory‐network reconstruction should often be interpreted as strong hypotheses rather than universally established causal mechanisms. Future studies integrating CRISPR‐based functional genomics, metabolite flux analysis, and biochemical validation will be essential for distinguishing correlation from causation and establishing robust genome‐to‐phenotype relationships.

Multiomics integration substantially improves mechanistic inference but does not by itself establish causality. The candidate regulatory mechanisms inferred through multiomics analyses require direct experimental validation, providing the rationale for the genome‐editing and synthetic‐biology approaches.

Collectively, multiomics integration has substantially improved reconstruction of regulatory pathways underlying specialized metabolism. However, transcript abundance, protein accumulation, enzyme activity, and metabolite levels are not always concordant, and many inferred regulatory interactions remain based primarily on correlation rather than direct experimentation. Integrating reverse genetics, biochemical assays, and genome editing will therefore be essential for validating these proposed regulatory models. A comparative summary of the current evidence base and remaining knowledge gaps is presented in (Table 6 **)**, providing a framework for prioritizing future mechanistic investigations.

**Table 6:** Comparative synthesis of the current mechanistic evidence linking genome architecture with specialized metabolism in medicinal and aromatic plants (MAPs).

Mechanistic feature	MAP examples discussed in this review	Current evidence base	Overall evidence strength	Knowledge gap
Tandem gene duplication	*Cannabis*, *Ocimum*, *Panax*	Comparative genomics, transcriptomics, metabolomics, partial functional validation	High	Functional characterization of duplicated genes across additional MAP species
Copy‐number variation (CNV)	*Cannabis*, *Panax*, *Capsicum*	Pangenomics, chemotype association, transcriptomics	Moderate–High	Direct causal validation using genome editing
Whole‐genome duplication (WGD)	*Panax*, *Ocimum*	Comparative genomics and evolutionary analyses	Moderate	Functional assessment of retained duplicated biosynthetic genes
Transposable‐element (TE)‐mediated regulation	*Artemisia*, *Cannabis*, *Papaver*	Comparative genomics, epigenomics, expression analyses	Moderate	Experimental validation of TE‐dependent regulatory mechanisms
Epigenomic regulation	*Cannabis*, *Artemisia*	DNA methylation, chromatin accessibility, transcriptomics	Emerging	Integration with functional genomics and metabolite‐flux analyses
Multi‐omics regulatory networks	*Artemisia*, *Panax*, *Salvia*	Integrated transcriptomics, proteomics, metabolomics	Moderate–High	Experimental validation of predicted regulatory interactions
Genome editing (CRISPR/Cas)	Selected MAP species	Functional genomics and targeted pathway engineering	High	Broader application to diverse MAP taxa

*Note:* Evidence grading: High = supported by functional validation (e.g., CRISPR/Cas, transgenic complementation, enzyme assays, or biochemical characterization); moderate‐high = supported by multiple independent genomic and multiomics studies with limited functional validation; moderate = supported primarily by comparative genomics and transcriptomic/metabolomic associations; emerging = supported mainly by epigenomic, computational, or exploratory studies requiring further validation.

## 5. Genome Editing and Synthetic Biology for Advancing Phytochemical Innovation in MAPs

### 5.1. Genome Editing for Functional Validation and Pathway Modulation

The expanding availability of chromosome‐scale genomes and multiomics datasets has created a mechanistic bridge between gene discovery and functional manipulation in MAPs. CRISPR‐based editing now enables targeted interrogation of BGCs, transcriptional regulators, and promoter elements that control metabolic flux through specialized metabolism pathways. These mechanistic interventions are particularly valuable for MAPs, where long generation times, polyploidy, and tissue‐specific metabolite localization have traditionally limited genetic improvement [41, 42]. Importantly, genome architecture revealed by long‐read assemblies including tandem arrays, paralog divergence, and regulatory hotspots directly informs CRISPR target selection. This marks a transition from descriptive gene catalogs to rational pathway engineering grounded in structural genomics.

Importantly, genome editing provides direct experimental validation of mechanistic hypotheses generated through genomics and multiomics analyses by testing whether targeted perturbation of specific structural variants, regulatory loci, or biosynthetic genes produces predictable metabolite phenotypes.

Beyond classical knockout strategies, emerging tools such as promoter editing, base editing, and multiplex CRISPR systems allow fine‐scale modulation of enzyme activity or pathway flux without disrupting upstream regulatory networks. Such allele‐level tuning is particularly relevant for BGCs, which often include tightly coregulated enzymes whose dosage balance determines metabolite yield. Integration of CRISPR with transcriptomic and epigenomic datasets further enables targeting of transcription factors, cis‐regulatory elements, and chromatin accessibility sites that coordinate pathway induction for example, trichome‐specific enhancers of diterpenoid and sesquiterpenoid biosynthesis. Collectively, these approaches move MAP biotechnology toward mechanism‐informed metabolic engineering.

An important limitation in many current MAP genome‐editing studies is the insufficient use of experimental controls required for robust mechanistic interpretation. For example, tissue‐specific negative controls and nonedited regeneration controls are often lacking in CRISPR/Cas experiments targeting specialized‐metabolism pathways, complicating interpretation of metabolite changes caused by developmental variation or transformation‐associated stress. Similarly, comprehensive off‐target validation through whole‐genome resequencing or high‐depth amplicon sequencing remains infrequently implemented in polyploid or highly repetitive MAP genomes. Future studies should incorporate tissue‐matched controls, independent edited lines, and quantitative metabolomic replication to strengthen causal inference between genome editing and phytochemical phenotype.

### 5.2. Synthetic Biology and Heterologous Pathway Reconstruction

In parallel, synthetic biology offers complementary strategies by reconstructing plant metabolic pathways in heterologous microbial hosts. Whereas genome editing experimentally perturbs endogenous plant pathways to validate mechanistic hypotheses in planta, synthetic biology reconstructs and optimizes these pathways in heterologous systems to enable scalable metabolite production and modular pathway engineering. Seminal work refactoring the artemisinin pathway in *Saccharomyces cerevisiae* established principles for modular design, enzyme balancing, and precursor channeling [43, 44]. Subsequent efforts have extended these frameworks to benzylisoquinoline alkaloids, cannabinoids, and terpenoids [45]. These microbial platforms bypass agricultural limitations and allow systematic testing of variant enzymes, transporters, or cofactor dependencies that are difficult to manipulate in planta. Mechanistically, they also reveal rate‐limiting steps or bottlenecks that can be targeted in MAPs using genome editing thus closing the loop between discovery and engineering.

**Figure 4:**
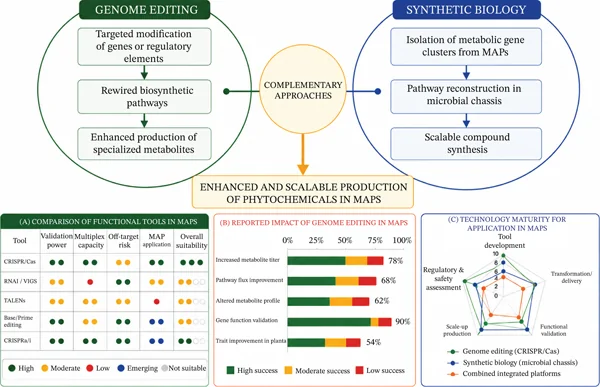
Complementary applications of genome editing and synthetic biology for engineering specialized metabolism in medicinal and aromatic plants (MAPs). Genome editing optimizes native biosynthetic pathways, whereas synthetic biology enables pathway reconstruction for scalable phytochemical production. Comparative panels summarize functional performance, reported applications, and technology maturity. (The figure created using https://www.biorender.com).

As summarized in (Table 7 **)**, genome editing validates causative metabolic genes uncovered through genomics and multiomics analyses, whereas synthetic biology converts these insights into scalable biosynthetic routes. (Figure 4 **)** conceptualizes this continuum: plant‐based editing enables precise rewiring of endogenous pathways and regulatory networks, while microbial chassis provide modular, evolvable platforms for reconstructing and optimizing BGCs outside the plant cellular context. Increasingly, AI‐driven design tools such as predictive gRNA scoring, enzyme‐function prediction, and pathway flux modeling are being integrated into both sides of this continuum, accelerating the transition from sequence information to engineered phytochemical phenotypes.

**Table 7:** Mechanistic applications of genome editing and synthetic biology in medicinal and aromatic plants (MAPs).

Plant/System	Genome editing/Synthetic biology approach	Targeted pathway or mechanistic goal	Mechanistic insight/outcome	Evidence strength	Reference
*Catharanthus roseus*	CRISPR/Cas9 knockout + promoter/regulatory edits	Monoterpenoid indole alkaloid (MIA) pathway	Validated rate‐limiting steps; revealed transcriptional–enzymatic coordination shaping alkaloid profiles	Strong (direct genome‐editing validation)	[41]
*Salvia miltiorrhiza*	CRISPR‐mediated promoter tuning and gene edits	Tanshinone (diterpenoid) biosynthesis	Identified flux‐controlling nodes; demonstrated how cis‐regulatory variation modulates diterpenoid accumulation	Strong (functional promoter and pathway validation)	[18]
*Cannabis sativa*	AI‐assisted gRNA design; epigenetic targeting concepts	Cannabinoid biosynthesis	Showed that structural variation + epigenomic state influence cannabinoid synthase expression and editing accessibility	Moderate (predictive and association‐based support; limited direct editing validation)	[40]
*Saccharomyces cerevisiae* (yeast)	Pathway refactoring (mevalonate → artemisinic acid)	Artemisinin precursor biosynthesis	Elucidated enzyme‐balancing and cofactor requirements; enabled industrial‐scale precursor production	Strong (experimentally reconstructed and validated pathway)	[43, 44]
Yeast/*E. coli*	Modular reconstruction of benzylisoquinoline alkaloid pathways	Morphinan and BIQ alkaloid intermediates	Demonstrated de novo microbial production; revealed transferrable pathway modules and bottlenecks for redesign	Strong (direct synthetic biology and pathway reconstruction evidence)	[45]

Despite these advances, significant challenges remain. Editing efficiency and regeneration remain low in many recalcitrant MAP species, particularly polyploids and species with limited transformation systems. Chromatin accessibility and epigenetic silencing further constrain editing outcomes in specialized tissues such as glandular trichomes. In synthetic systems, incomplete knowledge of plant enzyme complexes, cofactor requirements, or transport modules limits full pathway portability. Addressing these bottlenecks will require deeper tissue‐resolved omics, improved plant chassis with enhanced transformation competence, and iterative design‐build‐test cycles guided by metabolic modeling.

Ethical and regulatory considerations also shape the trajectory of genome editing and synthetic biology in MAPs. As detailed in (Box 1), regulatory frameworks differ internationally, and benefit‐sharing requirements under the Nagoya Protocol are particularly pertinent for biodiversity‐rich MAP species. Ensuring transparent governance and equitable access will be essential as genome‐engineered MAPs and microbially produced phytochemicals advance toward commercialization.

**Box 1: Ethical and regulatory considerations in genome editing and synthetic biology of MAPs**
The expanding application of genome editing and synthetic biology in MAPs raises several ethical and regulatory challenges. First, biosafety and containment are essential when transferring plant metabolic pathways into microbial hosts, particularly for pharmacologically active compounds; production platforms must comply with national biosafety regulations and Good Manufacturing Practice (GMP) standards [44]. Second, regulatory definitions of genome‐edited plants vary globally. Gene‐edited plants lacking foreign DNA are exempt from GMO regulations in countries such as the United States, Japan, and India, whereas the European Union classifies all CRISPR‐derived organisms as genetically modified [46]. Third, intellectual‐property frameworks and equitable benefit‐sharing are critical for MAPs originating from biodiversity‐rich regions. Compliance with the Nagoya Protocol is necessary for germplasm access and commercial development, and collaborative models involving local communities promote ethical innovation [47]. Finally, transparency in genome‐editing research including open data practices, traceable labeling, and public engagement is essential to ensure that technological advances do not compromise biodiversity, cultural heritage, or public trust.

Together, genome editing and synthetic biology are transforming MAP research from descriptive genomics into predictive and programmable phytochemistry. By integrating structural genomic insights with precision editing, pathway refactoring, and computational design, the field is progressing toward sustainable, mechanistically informed production of high‐value metabolites. (Table 7) distinguishes genome‐editing approaches aimed at causal functional validation in MAPs from synthetic‐biology platforms designed for heterologous pathway reconstruction and metabolic optimization.

Genome editing will be essential for validating candidate regulatory mechanisms predicted by multiomics analyses. AI‐based computational approaches now extend these experimentally validated datasets to predict regulatory interactions and optimize metabolic engineering strategies. An immediate priority is the systematic CRISPR validation of structural variants identified through pangenomic analyses, enabling direct testing of candidate regulatory mechanisms proposed in this review.

## 6. AI‐Driven Predictive Modeling and Computational Genomics in MAPs

AI and computational genomics now offer transformative capabilities for interpreting these multilayered datasets, enabling mechanistic inference from genome architecture to metabolic phenotype [48, 49]. AI prioritizes candidate regulatory nodes for experimental investigation rather than directly identifying causal regulators. Whereas Section 4 emphasizes biological interpretation of regulatory and metabolic processes through multiomics integration, this section focuses specifically on AI‐driven computational frameworks that automate pattern recognition, predictive modeling, BGC prioritization, and engineering‐target identification across large‐scale MAP datasets.

Applications of AI in MAP research increasingly revolve around resolving the structural and functional complexity of specialized metabolism [50]. Deep‐learning systems such as DeepBGC and PlantBGC Finder can identify BGCs even when they occur in repeat‐rich regions, lack canonical domain architecture, or vary among pangenome accessions conditions common in MAP species where lineage‐specific metabolites undergo rapid innovation [48, 51]. At the genomic level, AI‐enhanced variant callers (DeepVariant) significantly improve SNP and structural variant detection in highly heterozygous or polyploid MAP genomes, providing more accurate genetic markers for metabolite association studies and breeding programs [52]. These advances reframe genome architecture from a static catalog of genes to a dynamic map of evolutionary and metabolic potential.

AI‐assisted models provide predictive frameworks that require experimental validation before biological interpretation. BGC prediction additionally require clearly defined confidence parameters to ensure reproducibility. Deep‐learning BGC prediction platforms such as DeepBGC and PlantBGC Finder typically apply probability/confidence thresholds above 0.7–0.9 for high‐confidence cluster identification, whereas variant‐calling pipelines commonly incorporate minimum sequencing depths ≥ 20× and quality‐score filtering to improve structural‐variant accuracy in polyploid MAP genomes. Machine‐learning chemotype prediction models are generally evaluated using cross‐validation approaches and performance metrics including area under the curve (AUC), F1‐score, and prediction accuracies exceeding 80%–90% in well‐curated datasets.

AI has proven particularly powerful in linking genomic variation with metabolic output, a central challenge in MAP systems where chemotype diversity often arises from subtle allelic shifts, regulatory modifications, or CNV. Machine‐learning models trained on genomic and transcriptomic profiles have demonstrated strong predictive power for chemotype classification in *C. sativa*, where structural variation surrounding cannabinoid synthase loci shapes the production of THC, CBD, and related metabolites [12]. Similar AI‐supported analyses in *O. basilicum* identified terpenoid biosynthesis modules whose regulatory connectivity and expression patterns correspond to essential‐oil phenotypes, illustrating how AI can uncover mechanistic relationships obscured by gene‐family redundancy and environmental variability [36]. Across MAPs, such approaches reveal the architecture of metabolic regulation rather than simply cataloging molecular components.

These AI‐assisted frameworks provide powerful hypothesis‐generation platforms by integrating structural variation, chromatin features, coexpression networks, and metabolite profiles to prioritize candidate regulatory nodes associated with pathway activity and chemotype differentiation. However, the inferred regulatory relationships remain predictive and require experimental validation through functional genomics, genome editing, or biochemical assays before causal mechanisms can be established.

Beyond metabolic prediction, AI is rapidly becoming integral to genome editing and synthetic biology in MAPs. Tools such as DeepCRISPR incorporate sequence context, chromatin accessibility, and off‐target modeling to design guide RNAs optimized for editing efficiency and specificity in complex genomes [42]. AI‐driven structural predictors (AlphaFold2) and protein‐language models (ProtCNN) accelerate functional annotation of the numerous uncharacterized enzymes in MAP metabolic pathways, enabling rational engineering of enzymes with improved catalytic activity, altered substrate specificity, or optimized domain organization [48, 53]. These capabilities shorten the design‐build‐test cycle for metabolic pathway rewiring in planta and for pathway refactoring in microbial hosts.

AI′s growing role in synthetic biology is equally transformative. Machine‐learning‐integrated metabolic models such as RePath and OptFlux‐ML support automated in silico reconstruction of biosynthetic pathways, prediction of rate‐limiting steps, and design of optimized enzyme combinations for heterologous production systems [54]. When combined with multiomics data, these models allow in planta and microbial platforms to be engineered from first principles, increasing yield, stability, and pathway modularity. Reproducibility and cross‐study comparability of AI‐assisted, pangenomic, and multiomics workflows additionally depend on standardized analytical parameters including GWAS significance thresholds, coexpression cutoffs, sequencing depth, BGC confidence scores, and machine‐learning validation metrics. Analytical criteria commonly applied across MAP genomic studies are summarized in (Table 8).

**Table 8:** Analytical parameters commonly used in MAP genomics, pangenomics, multi‐omics, and AI workflows.

Workflow	Common analytical parameter	Typical threshold/range	Purpose
GWAS	Minor allele frequency (MAF)	> 0.05	Reduce rare‐variant noise
GWAS	Genome‐wide significance	10^−5^–10^−8^	Association confidence
QTL mapping	LOD score	> 3.0	Significant QTL detection
WGCNA	Pearson correlation	> 0.7–0.9	Coexpression module definition
Differential expression	Log₂ fold change	≥ 1–2	Significant gene‐expression shifts
Differential expression	Adjusted *p*‐value/FDR	< 0.05	Statistical confidence
BGC prediction	DeepBGC confidence score	> 0.7–0.9	High‐confidence cluster detection
Variant calling	Sequencing depth	≥ 20×	SV/SNP accuracy
AI prediction	Cross‐validation accuracy	> 80–90%	Model robustness
Metabolomics	Biological replicates	≥ 3	Experimental reproducibility

As summarized in (Table 9 **)**, AI applications now span gRNA design, variant calling, BGC prediction, metabolite modeling, enzyme function inference, and computational pathway design. Together, these tools provide a mechanistic continuum from gene discovery to engineering.

**Table 9:** AI and computational approaches applied to medicinal and aromatic plants.

Tool/Approach	Application in MAPs	Key outcome/Insight	Reference
DeepCRISPR	AI‐based guide RNA design for CRISPR genome editing	Optimized gRNA selection and minimized off‐targets in polyploid MAP genomes	[42]
DeepBGC/PlantBGC Finder	Deep‐learning prediction of biosynthetic gene clusters	Discovery of cryptic or non‐canonical BGCs in *Ocimum*, *Cannabis*, and *Papaver*	[48, 51]
ML‐based metabolite prediction (Random Forest, CNNs)	Chemotype‐genotype modeling in *Cannabis sativa* and *Ocimum basilicum*	Accurate prediction of metabolite composition from genomic and transcriptomic data	[12, 36]
AI‐assisted network prioritization frameworks	Multiomics network inference	Computational prioritization of regulatory hubs and metabolite‐associated pathway candidates from large‐scale omics datasets	[9, 55]
DeepVariant/DeepAnnotator	AI‐assisted genome assembly and variant calling	Improved SNP and structural‐variant accuracy in complex plant genomes	[49, 52]
AI‐assisted enzyme function prediction (ProtCNN, AlphaFold2)	Predicting enzyme specificity and catalytic residues in MAP biosynthetic enzymes	Accelerated annotation of uncharacterized oxidoreductases and synthases	[48, 53]
AI‐integrated metabolic pathway design (RePath/OptFlux‐ML)	In silico pathway reconstruction for synthetic biology applications	Automated design of optimized biosynthetic routes for phytochemical production	[49, 54]

**Figure 5:**
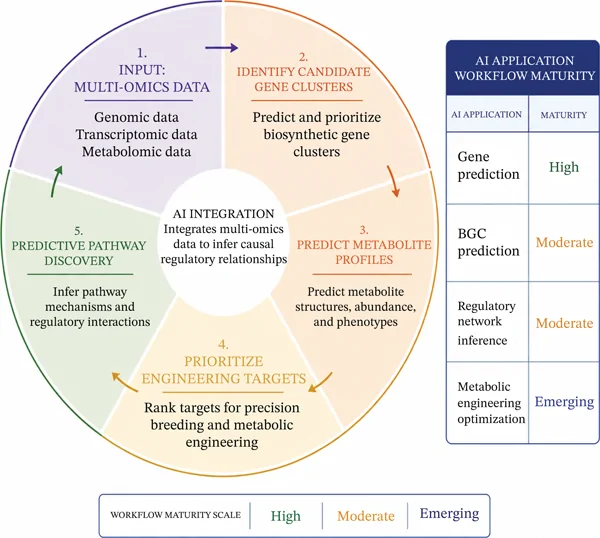
Artificial intelligence integrates genomic, transcriptomic, and metabolomic data to identify candidate biosynthetic gene clusters, predict metabolite profiles, and prioritize engineering targets, enabling predictive pathway discovery and precision improvement of medicinal and aromatic plants. The workflow additionally illustrates how computational and multi‐omics data can be integrated to infer causal regulatory relationships underlying metabolite phenotypes. (Created using https://www.biorender.com).

This conceptual workflow is captured in (Figure 5 **)**, which diagrams how multiomics datasets flow into an AI‐based analytical engine that outputs candidate BGCs, regulatory modules, predictive chemotype models, and engineering targets for genome editing or synthetic biology. By transforming data into actionable hypotheses, AI shifts MAP metabolic research from descriptive exploration to predictive, hypothesis‐driven design.

Looking ahead, AI has considerable potential to reshape MAP biotechnology through the development of explainable AI systems capable of improving the biological interpretation of computational predictions. However, realizing this potential will depend on the availability of large, experimentally validated training datasets, transparent model architectures, standardized benchmarking frameworks, and close integration with functional genomics and biochemical validation. Collectively, these innovations position AI as a central driver of the next generation of MAP research, enabling deeper mechanistic insight, faster pathway discovery, and more sustainable phytopharmaceutical development.

Importantly, AI‐generated pathway predictions and regulatory inferences should be interpreted as hypothesis‐generating frameworks unless supported by direct experimental validation, biochemical characterization, or genome‐editing evidence. Moreover, AI‐driven pathway prediction studies frequently lack standardized benchmarking datasets and experimentally validated negative controls, making cross‐platform comparison difficult. Future AI‐assisted MAP workflows will benefit from integrating curated validation datasets, experimentally confirmed pathway annotations, and transparent reporting of model‐performance metrics.

AI has substantially improved prioritization of candidate genes, regulatory modules, and biosynthetic pathways in MAP research. Nevertheless, prediction accuracy varies among species and datasets, and computational models do not necessarily distinguish causal biological mechanisms from statistical associations. Consequently, AI‐derived predictions should be interpreted as hypothesis‐generating tools requiring independent experimental validation.

## 7. Conservation Genomics and Sustainable Utilization of MAPs

The remarkable biochemical diversity of MAPs originates from deep reservoirs of genetic variation shaped by evolutionary history, environmental adaptation, and ecological interactions. Yet this diversity is increasingly at risk. Rapid habitat transformation, overharvesting, climate change, and unregulated commercial exploitation have accelerated genetic erosion in many culturally and economically important MAP species [21, 56]. As global demand for plant‐derived pharmaceuticals grows, ensuring the resilience and equitable use of MAP resources requires a transition from reactive conservation to genomically informed, predictive, and sustainability‐driven management. Conservation genomics now empowered by long‐read sequencing, pangenomics, multiomics profiling, and AI provides an unprecedented foundation for this transition [11, 56].

High‐quality genomes and multiaccession datasets have transformed our capacity to detect the evolutionary and functional dimensions of MAP diversity. Reference genomes, transcriptomes, and especially pangenomes reveal population‐level variation in BGCs, adaptive alleles, and structural variants linked to ecological gradients. Such insights refine conservation units far beyond morphological or geographic criteria. For example, genome‐wide SNPs and haplotypes have resolved fine‐scale population structure in *Rauvolfia serpentina* and *Withania somnifera*, clarifying which populations harbor rare alleles for alkaloid biosynthesis or stress resilience and should therefore be prioritized for ex situ conservation or restoration programs [20, 57]. These resources also guide the selection of genetically accessions for seed banks and living collections, strengthening the foundation for long‐term genetic safeguarding.

Landscape genomics extends this approach by coupling genomic variation with ecological and climatic parameters to identify drivers of local adaptation. This integrative lens reveals not only where genetic diversity resides but why it persists. In *A. annua*, landscape genomic models have identified environmental gradients shaping allelic distributions associated with artemisinin content and temperature tolerance, enabling predictive maps of climate‐vulnerable and resilient populations [16]. Similar analyses in *P. ginseng* and *Curcuma longa* illuminate adaptive signatures linked to phytochemical stability, guiding in situ protection of genetically robust populations that are likely to withstand climate stress [17, 21]. These insights support evidence‐based decisions about habitat prioritization, translocation, assisted gene flow, and climate‐smart conservation planning.

The shift to genomically informed conservation is bolstered by rapidly expanding digital biobanking infrastructure. Global repositories such as the Medicinal Plant Genome Atlas (MPGA) and the Global Genome Biodiversity Network (GGBN) integrate genomic, metabolomic, ecological, and voucher‐linked metadata, providing standardized, interoperable platforms for long‐term preservation of MAP genetic information [56]. When paired with AI‐driven predictive models (as described in the preceding section), these resources can forecast population decline, identify genomic bottlenecks, and simulate outcomes of conservation interventions offering decision‐makers a powerful toolkit for risk assessment and management. Regional initiatives such as the India Medicinal Plant Genomics Consortium further suggest how coordinated genomic surveillance can support sustainable‐use policies and breeding programs tailored to national biodiversity contexts [21]. (Table 10 **)** summarizes initiatives linking genomic and digital frameworks for MAP conservation and sustainable utilization.

**Table 10:** Genomic and digital frameworks supporting conservation and sustainable utilization of medicinal and aromatic plants (MAPs).

Initiative/Platform	Data types/Functional scope	Mechanistic contribution to conservation and sustainable use	MAP species	Reference
Medicinal Plant Genome Atlas (MPGA)	High‐quality genomes, metabolomes, ecological and voucher metadata	Identifies adaptive alleles and chemotype‐associated variants; supports genomic monitoring and selection of core germplasm for conservation and cultivation	*Withania somnifera*, *Ocimum basilicum*	[56]
Global Genome Biodiversity Network (GGBN)	Voucher‐linked genome sequences, metadata standards, global repository	Ensures traceable, high‐quality genomic resources for comparative conservation genomics; enables cross‐institutional data integration	Multiple MAP lineages	[56]
India Medicinal Plant Genomics Consortium	Regional genome assemblies, SNP panels, population datasets	Guides conservation‐oriented breeding; identifies population structure, rare alleles, and genomic erosion hotspots for national MAP species	*Rauvolfia serpentina*, *Curcuma longa*	[21]
Plant Adaptome Project	Landscape genomics, climate gradients, environmental association models	Predicts genomic vulnerability and climate‐resilient alleles; informs restoration, habitat prioritization, and assisted gene flow strategies	*Artemisia annua*, *Panax ginseng*	[16]
Nagoya‐Compliant Digital Access and Benefit‐Sharing Framework	Legal metadata, digital sequence information (DSI) governance tools	Ensures ethical access, benefit‐sharing, and compliance for MAP genetic resources; supports equitable use in breeding and biotechnology	Global MAP species	[47]

**Figure 6:**
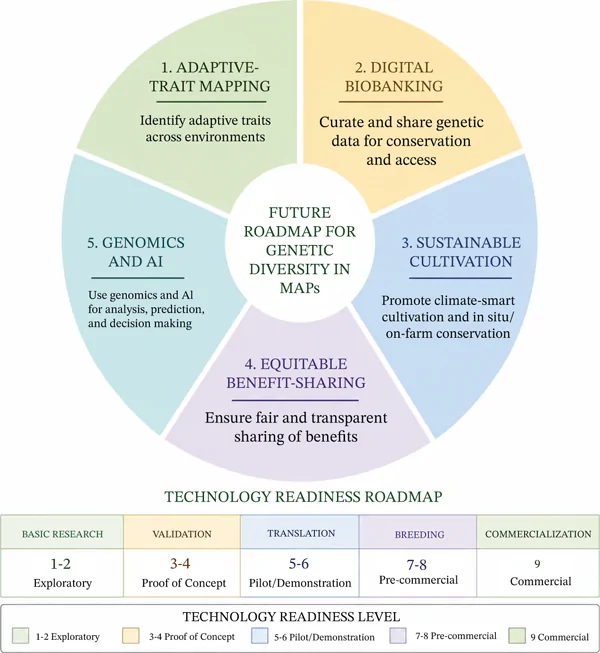
Future roadmap for genomics‐enabled conservation of medicinal and aromatic plants (MAPs). The figure integrates genomics, AI, digital biobanking, sustainable cultivation, and equitable benefit sharing with a technology‐readiness roadmap linking basic research to commercialization. (Created using https://www.biorender.com).

Importantly, conservation genomics is not segregated from innovation; it is a prerequisite for sustainable biotechnological development. Genomic insights identifying alleles linked to yield, resilience, or specialized‐metabolite biosynthesis directly inform molecular domestication strategies, enabling cultivation of high‐value MAPs and reducing harvesting pressure on wild populations [40]. This integrative perspective is particularly relevant for food‐medicine homologous MAP resources, whose nutritional and pharmacological functions are increasingly recognized in chronic disease management, including antioxidant protection and lipid‐metabolism regulation [1]. Figure 6 illustrates this integrative paradigm: biodiversity monitoring and genomic characterization feed into AI‐assisted prediction, breeding, and genome editing pipelines, which in turn reduce reliance on overexploited wild stocks while preserving phytochemical fidelity. This feedback loop the reciprocal reinforcement of conservation and innovation provides a roadmap for aligning technological advancement with ecological stewardship.

Despite these advances, substantial challenges remain. Many MAPs remain genomically underrepresented, particularly those from tropical biodiversity hotspots where data sovereignty, access and benefit‐sharing (ABS) regulations, and technical limitations restrict sequencing efforts. This challenge is particularly relevant for highly threatened medicinal orchids (Orchidaceae) and endemic Rutaceae taxa, many of which possess unique phytochemical repertoires but remain genomically underrepresented despite increasing conservation concern.

Translating genomic data into management action requires cross‐sector collaboration, long‐term funding, transparent data governance, and ethical frameworks that acknowledge indigenous knowledge and prior rights. Integrating genomic innovation with climate modeling, AI‐based forecasting, and equitable benefit‐sharing policies will be critical to ensuring that the next decade of MAP research supports both global health needs and the resilience of plant biodiversity.

Collectively, these limitations indicate that future mechanistic studies in MAPs will require more rigorous experimental validation frameworks integrating appropriate controls, replicated datasets, chromatin‐state verification, and functional perturbation analyses. Although genomics, multiomics, AI, and genome‐editing technologies have substantially accelerated pathway discovery, inconsistencies in validation strategies continue to limit reproducibility and mechanistic confidence across MAP systems. Key methodological gaps and recommended validation priorities for future MAP functional‐genomics research are summarized in (Box 2).

**Box 2:** Experimental and validation priorities for mechanistic MAP genomics.

Research area	Frequently missing controls	Recommended future validation
CRISPR editing	Tissue‐matched negative controls	Independent edited lines + off‐target sequencing
Epigenomics	Chromatin‐state validation	ATAC‐seq + ChIP‐seq + reporter assays
GWAS/QTL	Environmental replication	Multilocation validation populations
Multiomics	Functional perturbation	Reverse genetics + enzyme assays
AI prediction	Benchmark datasets	Experimentally validated training/testing datasets

Collectively, the studies reviewed here support a mechanistic framework in which genome architecture influences phytochemical diversity through interconnected regulatory layers involving chromatin accessibility, transcriptional coordination, enzyme dosage, pathway modularity, and metabolic flux control. Thus, MAP metabolite phenotypes emerge not simply from gene presence or absence, but from dynamic interactions between structural genomic variation and regulatory network architecture. The revised framework distinguishes biologically interpretive multiomics approaches from AI‐driven predictive and engineering‐oriented computational workflows, thereby reducing methodological redundancy while strengthening mechanistic continuity across MAP genomic research.

### 7.1. Outstanding Mechanistic and Translational Challenges in MAP Genomics

Despite major advances in long‐read sequencing, pangenomics, multiomics integration, AI‐assisted prediction, and genome engineering, several unresolved mechanistic and translational challenges continue to constrain progress in MAP research. Although current studies increasingly identify structural variation, BGCs, and metabolite‐associated loci, the causal regulatory mechanisms linking genome architecture with specialized‐metabolism phenotypes remain insufficiently understood.

A major bottleneck is the limited accuracy and experimental validation of current plant BGC‐prediction tools. Existing frameworks often struggle with lineage‐specific metabolic architectures, TE‐rich genomic regions, and poorly annotated nonmodel MAP genomes, leading to false positives and incomplete cluster resolution. Improving prediction reliability will require experimentally validated training datasets, lineage‐aware algorithms, chromatin‐accessibility integration, and functional perturbation analyses.

Another key challenge involves the epigenetic regulation of tissue‐specific metabolism. Although chromatin accessibility, DNA methylation, and histone modifications are increasingly associated with pathway activation, the regulatory mechanisms governing metabolite biosynthesis in specialized tissues such as glandular trichomes and roots remain poorly resolved. Future progress will depend on integrating single‐cell epigenomics, spatial transcriptomics, ATAC‐seq, and targeted epigenome editing to establish causal regulatory relationships.

Polyploid MAP species present additional complexity because the functional differentiation of duplicated biosynthetic genes remains largely undefined. WGD expands TPSs, cytochrome P450s, glycosyltransferases, and regulatory gene families, yet the mechanisms governing subfunctionalization, neofunctionalization, dosage partitioning, and epigenetic divergence of paralogs remain unclear, particularly in highly polyploid species such as *P. ginseng* and *H. tuberosus*.

Finally, major translational barriers continue to limit the conversion of genomic discoveries into successful metabolic‐engineering applications. Many MAPs remain recalcitrant to transformation and regeneration, whereas heterologous pathway reconstruction often fails to reproduce native metabolite yields because of an incomplete understanding of transport systems, compartmentalization, cofactor balance, and regulatory coordination. Addressing these challenges will require integrated design‐build‐test frameworks combining functional genomics, genome editing, synthetic biology, and quantitative metabolic modeling.

Genomics‐assisted conservation provides powerful opportunities for preserving medicinal plant diversity; its implementation remains constrained by uneven genomic resources, limited sampling, and differences in regional conservation priorities. Consequently, conservation strategies successful in one lineage may not always be directly transferable to other medicinal plant groups.

## 8. Future Perspectives and Roadmap

The rapid expansion of high‐quality genomic resources, together with the methodological priorities summarized in (Box 2), has positioned MAP genomics for a transition from resource generation toward functional implementation. Over the next decade, progress will depend less on continued data accumulation alone and more on establishing experimentally validated frameworks that connect genome architecture with specialized‐metabolism phenotypes. Future MAP studies will additionally require greater standardization of analytical thresholds, benchmarking datasets, and reproducibility metrics to enable reliable cross‐study comparison and translational pathway engineering.

A primary short‐term priority (3–5 years) is the generation of complete, functionally annotated genomic landscapes across taxonomically diverse MAP lineages. Particular emphasis should be placed on expanding population‐level genomic resources for underrepresented medicinal families including Lamiaceae, Orchidaceae, Rutaceae, Apocynaceae, and Zingiberaceae to reduce taxonomic bias in models of specialized metabolism. A realistic milestone will be the establishment of chromosome‐scale assemblies, haplotype‐resolved genomes, and pangenomes for major MAP lineages to improve identification of BGCs, adaptive alleles, and structural variants associated with metabolic specialization. Future MAP genomics will likely transition toward graph‐based and super‐pangenome architectures capable of representing complex structural variation and accession‐specific metabolic loci more accurately than single linear reference genomes [34]. In parallel, advances in single‐cell sequencing, spatial transcriptomics, and organelle genomics will provide cell‐type and compartment‐specific insight into specialized‐metabolism pathways.

A second medium‐term priority (5–10 years) involves experimentally supported reconstruction of regulatory and metabolic networks controlling phytochemical diversity. Although structural variation, chromatin accessibility, and epigenetic regulation are increasingly associated with metabolite biosynthesis, many reported BGCs remain incompletely validated, and chemotype variation cannot always be explained by genomic variation alone. Future studies should therefore prioritize standardized multiomics pipelines combined with CRISPR/Cas validation, enzyme characterization, and metabolic assays to distinguish causal regulatory mechanisms from association‐based predictions. AI and machine‐learning platforms have considerable potential to model genotype‐to‐chemotype relationships, infer regulatory networks, and predict pathway bottlenecks [58]; however, progress over the next decade will require MAP‐specific training datasets, experimentally validated pathway databases, and interpretable algorithms. However, these computational frameworks must evolve toward interpretable and experimentally validated systems capable of guiding hypothesis‐driven experimentation rather than functioning solely as predictive black boxes.

A third long‐term priority (> 10 years) is the translation of validated genomic discoveries into scalable metabolic‐engineering platforms. CRISPR‐based tools including base editing, prime editing, and programmable transcriptional regulators will enable precise tuning of pathway genes and regulatory modules in planta [42, 59, 60]. Parallel advances in synthetic biology will support modular reconstruction of MAP pathways in microbial or plant chassis for climate‐independent phytochemical production. A long‐term milestone will be the development of predictable design‐build‐test‐learn platforms integrating AI‐guided enzyme selection, quantitative metabolic modeling, high‐throughput screening, and optimized chassis systems for precision phytochemical engineering.

To translate genomic resources into biological and translational outcomes, future research should follow four milestone‐oriented directions spanning short‐term discovery, medium‐term validation, and long‐term predictive engineering. First, structural variants identified through pangenomic analyses should be functionally validated using CRISPR/Cas‐based genome editing, reverse genetics, and biochemical assays to establish causal genome‐to‐metabolite relationships. Second, integration of chromosome‐scale pangenomes with single‐cell, spatial transcriptomic, epigenomic, and metabolomic datasets should be used to resolve cell‐type‐specific regulation of specialized metabolism, particularly within secretory tissues such as glandular trichomes and laticifers. Third, AI‐assisted predictive models should move beyond gene annotation to reconstruct regulatory networks, prioritize candidate biosynthetic genes, and predict pathway flux using experimentally validated multiomics datasets. Finally, future studies should establish standardized MAP pangenome resources, interoperable multiomics databases, and community benchmarking frameworks to improve reproducibility, comparative analyses, and translational applications in precision breeding, metabolic engineering, and sustainable phytochemical production.

Achieving these short, medium, and long‐term milestones will require coordinated integration of high‐quality pangenomes, functional validation pipelines, standardized multiomics resources, and predictive computational models. Such a strategy may enable MAP research to progress from descriptive genome characterization toward experimentally validated and predictive genomic frameworks supporting phytochemical discovery, precision breeding, and sustainable utilization.

Despite substantial advances, several important comparative questions remain unresolved. Most experimentally validated genome‐to‐metabolite relationships originate from a small number of model MAP species, limiting broader evolutionary inference. Whether mechanisms such as tandem duplication, TE‐mediated regulation, chromatin remodeling, and biosynthetic gene‐cluster evolution contribute similarly across phylogenetically distant medicinal plants remains uncertain. Expanding functional genomics, comparative pangenomics, and genome editing to underrepresented MAP families will therefore be essential for distinguishing universally conserved mechanisms from lineage‐specific innovations.

## 9. Conclusions

MAPs represent an extraordinary reservoir of biochemical diversity, yet fully harnessing their potential requires an integrated understanding of how genome architecture, regulatory networks, and evolutionary processes shape specialized metabolism. Current evidence supports an important contribution of genome architecture to specialized metabolism; however, experimentally validated mechanistic relationships remain available for only a limited number of MAP systems. Advances in long‐read sequencing, pangenomics, and multiomics have begun to resolve the complexity of MAP genomes, revealing how structural variation, duplication history, and BGC dynamics contribute to chemotype diversity and adaptive specialization. When coupled with AI, these datasets now provide predictive frameworks for identifying pathway genes, modeling metabolite phenotypes, and prioritizing targets for molecular intervention.

A defining feature of MAPs is that specialized metabolism evolves within highly dynamic genomic regions where structural variation, gene duplication, TE activity, and complex regulatory networks interact to generate extraordinary phytochemical diversity. Consequently, understanding MAP genomes requires mechanistic interpretation of genome architecture rather than simply increasing the number of available genome assemblies. Integrating structural genomics with functional validation will therefore remain the central challenge in translating genomic diversity into pharmacologically meaningful biological insight.

Collectively, current evidence suggests that mechanistic genomic insights are emerging across an increasingly diverse range of medicinal plant families, although genomic resources remain unevenly distributed among taxonomic lineages. Although several mechanistic hypotheses are consistently supported by comparative genomics and multiomics analyses, relatively few genome‐to‐metabolite relationships have been confirmed through direct functional validation. Future research should therefore prioritize resolving conflicting observations and experimentally distinguishing causal regulatory mechanisms from association‐based genomic relationships.

At the same time, genome editing and synthetic biology offer important opportunities to validate, optimize, and reconstruct plant metabolic pathways, enabling both in planta enhancement of phytochemical production and scalable microbial biosynthesis. These technologies form a continuum with conservation genomics, which ensures that the genetic resources underpinning innovation are identified, monitored, and protected amid accelerating environmental change and harvesting pressure. Importantly, these mechanistic principles are conserved across phylogenetically distant MAP groups from alkaloid‐rich *Papaver* and *Rauvolfia*, to terpenoid‐dominated *Artemisia*, *Salvia*, *Ocimum*, and *Mentha*, and phenolics‐rich systems such as *Curcuma*.

The emerging landscape of MAP research suggests that future progress will depend not on any single technology, but on the strategic integration of genomics, computation, engineering, and conservation. By uniting mechanistic insight with responsible stewardship, the field is poised to develop sustainable, equitable, and innovative solutions for phytopharmaceutical production. This holistic approach ensures that MAP biodiversity both genetic and metabolic continues to support human well‐being while preserving the evolutionary heritage of these essential plant species.

## Author Contributions

All authors contributed to the study conception and design. Writing—original draft preparation: Himanshu Saini. Writing—review and editing: Jyoti Yadav, Sahil Shamkuwar, and Shubham Singh. Conceptualization: Vishal Johar. Methodology: Vikrant Jaryan. Formal analysis and investigation: Shalu Vyas. Resources: Atin Kumar. Supervision: Anand Kumar. Deepak Nanda and Jeevan Jyoti Kaushik contributed equally to this work.

## Funding

No funding was received for this manuscript.

## Disclosure

All authors read and approved the final manuscript. All authors commented on previous versions of the manuscript.

## Conflicts of Interest

The authors declare no conflicts of interest.

## Data Availability

The data that support the findings of this study are available from the corresponding author upon reasonable request.
